# Supplementary material for: Conversational content is organized across multiple timescales in the brain
Source: Nat Hum Behav. 2025 Jun 11;9(10):2066–78. doi: 10.1038/s41562-025-02231-4 (PMC12545197; doi:10.1038/s41562-025-02231-4)
Supplement: Supplementary file 1 — Supplementary Figs. 1–19, Tables 1–5 and results. [file 41562_2025_2231_MOESM1_ESM.pdf]

nature human behaviour

Supplementary information https://doi.org/10.1038/s41562-025-02231-4

# Conversational content is organized across multiple timescales in the brain

In the format provided by the authors and unedited

# Supplementary Information


***Supplementary Results***

1. Removing head motion artifacts.
2. Removing low-level sensorimotor signals.

***Supplementary Figures***

1. Box plots of the mean number of speech samples per run across different conditions.
2. Box plots of head motion across fMRI volumes.
3. Cortical maps of Head Motion model prediction accuracy.
4. Cortical maps of prediction accuracy of random normal embeddings.
5. Cortical maps of prediction accuracy of additional low-level features.
6. Cortical maps of Separate Linguistic model prediction accuracy.
7. Cortical maps of cross-modality prediction accuracy.
8. Cortical maps of Unified Linguistic model prediction accuracy.
9. Prediction performance of linguistic models across context lengths and layers.
10. Variance explained by production, comprehension, and their intersection.
11. Weight correlation averaged across linguistic and cross-modal voxels.
12. Weight correlation between the Unified and Separate Linguistic model.
13. Weight correlation averaged across production, comprehension, and bimodal voxels.
14. Cortical maps of weight correlation across cross-modal voxels.
15. Cortical maps of weight correlation differences between the Unified and Separate Linguistic model.
16. Cortical maps of the best variance partition.
17. Cortical maps of weight correlation across bimodal voxels.
18. Replicated results using a base GPT model without instruction tuning.
19. The number of significant principal components (PCs) identified for each participant.

***Supplementary Tables***

1. Linear mixed effects model statistics for prediction accuracy.
2. Linear mixed effects model statistics for weight correlation.
3. Linear mixed-effects model statistics for weight correlation comparisons.
4. The number of fMRI data samples (or runs) collected per session.
5. Topic list used in the dialogue experiment.

## Supplementary Results

### 1. Removing head motion artifacts

We assessed head motion using framewise displacement (FD; Power et al., 2012) and compared FD between scans conducted with and without speech production (**Supplementary Fig. 2**). At the individual level, six participants showed increased head motion during speech production, with effect sizes ranging from small (Cohen's $d < 0.3$; P1, P3, P5, P7) to medium ($0.3 <$ Cohen's $d < 0.5$; P2, P6). In contrast, two participants (P4, P8) showed slightly greater head motion in the absence of speech production, both with small effect sizes (Cohen's $d < 0.3$). At the group level, no significant difference in head motion was observed between conditions (Mann-Whitney $U$ test, $U = 40$, $P = 0.44$). The FD during speech production was 0.239 ± 0.047 mm (mean ± standard deviation; range: 0.182–0.322 mm), compared to 0.219 ± 0.044 mm (range: 0.175–0.323 mm) in the absence of speech production.

To minimize motion-related artifacts in the BOLD responses, we regressed out six rigid-body motion parameters along with FD. Model performance, evaluated by the Pearson correlation between predicted and observed timeseries, was low to medium across the cortex (mean ± standard deviation of Fisher $z$-transformed correlation = 0.0566 ± 0.0099; range: 0.0386–0.0699; see **Supplementary Fig. 3** for individual cortical maps). These findings indicate that head motion can partially account for brain activity during conversation, with relatively high prediction accuracy in primary motor cortex. To isolate higher-level linguistic contributions directly related to conversational content, the motion-predicted timeseries was regressed out in subsequent analyses.

### 2. Removing low-level sensorimotor signals

After removing artifacts caused by head motion, we further removed low-level sensorimotor contributions—including those associated with speech articulation and auditory perception—that are not directly related to the linguistic processing of words and sentences. To model these low-level signals, we employed random normal embeddings (Zada et al., 2024) with the same dimensionality (2,816 dimensions) as GPT. Voxel-wise modeling was then conducted on the BOLD responses after regressing out head motion effects. This model achieved high prediction accuracy in regions associated with articulatory-motor and auditory systems (mean ± standard deviation of Fisher $z$-transformed correlation = 0.0720 ± 0.0112; range: 0.0580–0.0944; see **Supplementary Fig. 4** for individual cortical maps).

To further validate that the random normal embeddings captured low-level sensorimotor signals, an additional low-level sensorimotor model was fit to the residual BOLD responses using explicit features. These included syllable and morpheme counts per utterance, as well as auditory features derived from a cochleargram based on Lyon's passive cochlea model (https://github.com/theunissenlab/tlab/tree/master/AuditoryToolbox), which employs logarithmically spaced filters with a bandwidth defined by

$$BW = \frac{\sqrt{cf^2 + ebf^2}}{Q}$$

where $cf$ is the characteristic frequency, $ebf$ is the ear break frequency (set to 1,000 Hz), and $Q$ is the quality factor (set to 8). This cochlear filter bank produced 80 frequency channels spanning 264–7,630 Hz, spaced at 25% of their respective bandwidths. To match the Nyquist frequency of fMRI data (0.5 Hz), we applied a low-pass filter followed by a Lanczos filter. The resulting low-level model yielded very low prediction accuracy (mean ± standard deviation of Fisher $z$-transformed correlation = 0.0137 ± 0.0074; range: 0.0048–0.0268; see **Supplementary Fig. 5** for individual cortical maps). These results confirm that the random normal embedding model had already accounted for a substantial potion of the variance related to basic sensory and motor processes. Therefore, for the main analyses, we used BOLD responses after regressing out both head motion artifacts and predictions from the random normal embedding model.

## Supplementary Figures

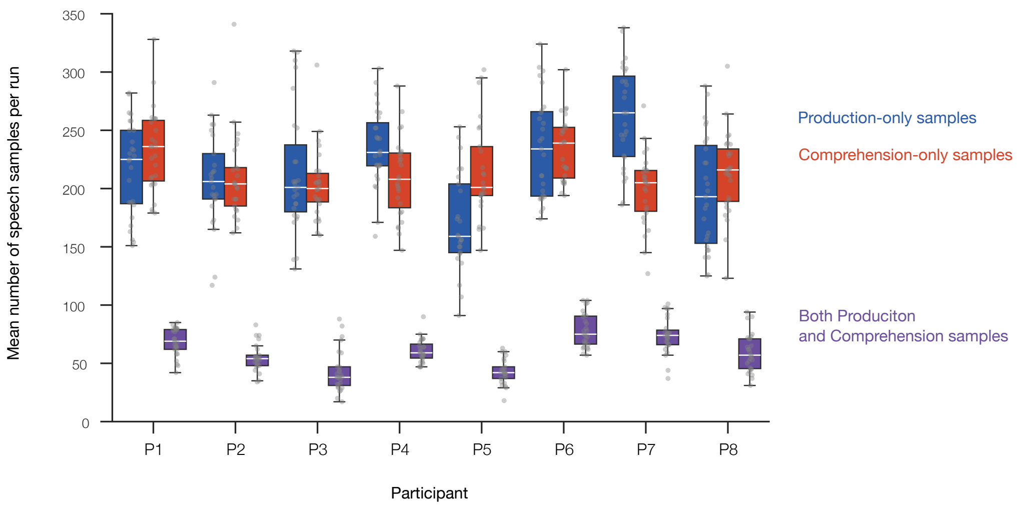


**Supplementary Fig. 1 | Box plots of the mean number of speech samples per run across different conditions.** In the Production-only and Comprehension-only samples, participants either produced or perceived speech, respectively. In the both Production and Comprehension samples, participants simultaneously produced and perceived speech within a single fMRI volume. The central line in each box plot represents the median, while the box boundaries show the upper and lower quartiles. Whiskers extend to 1.5 times the interquartile range. Individual data points represent the mean values per run.

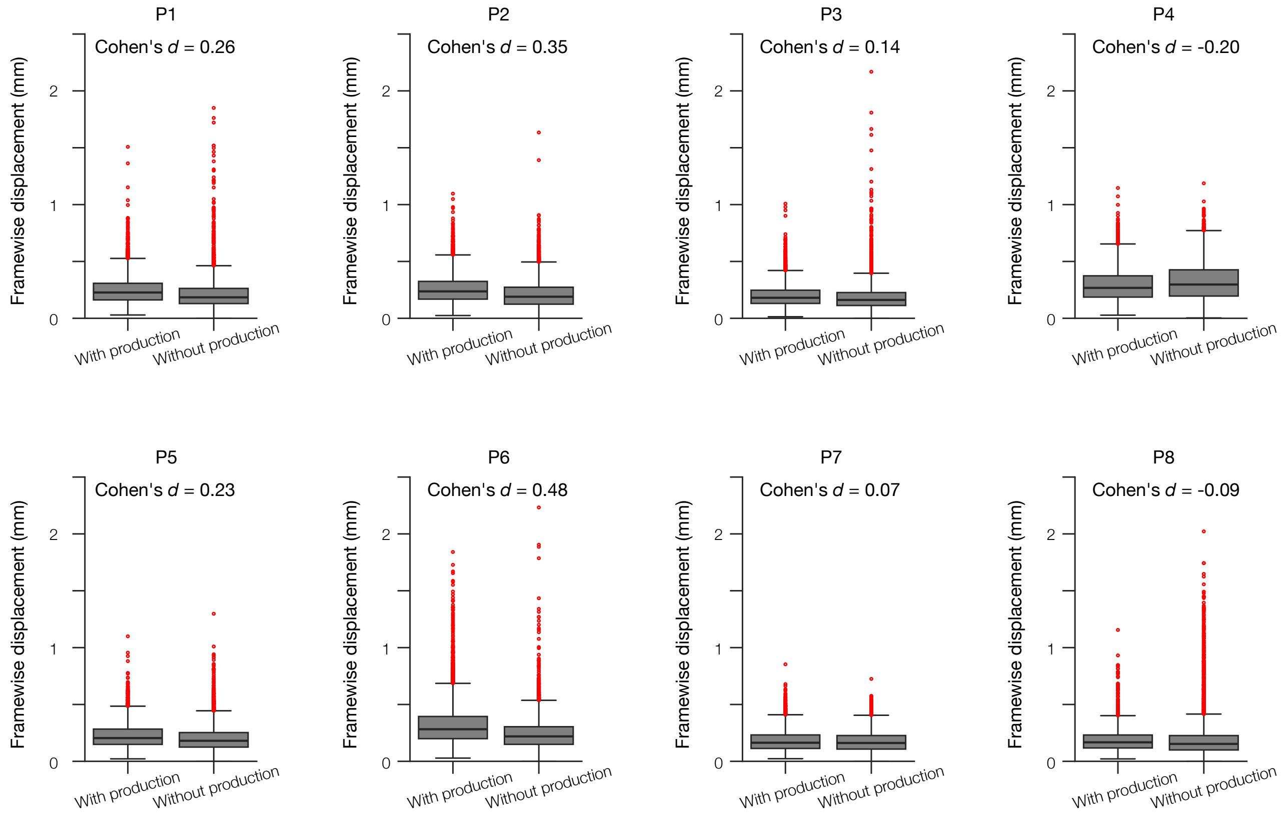


**Supplementary Fig. 2 | Box plots of head motion across fMRI volumes.** The central line in each box plot represents the median, while the box boundaries show the upper and lower quartiles. Whiskers extend to 1.5 times the interquartile range. Outliers are marked with red circles.

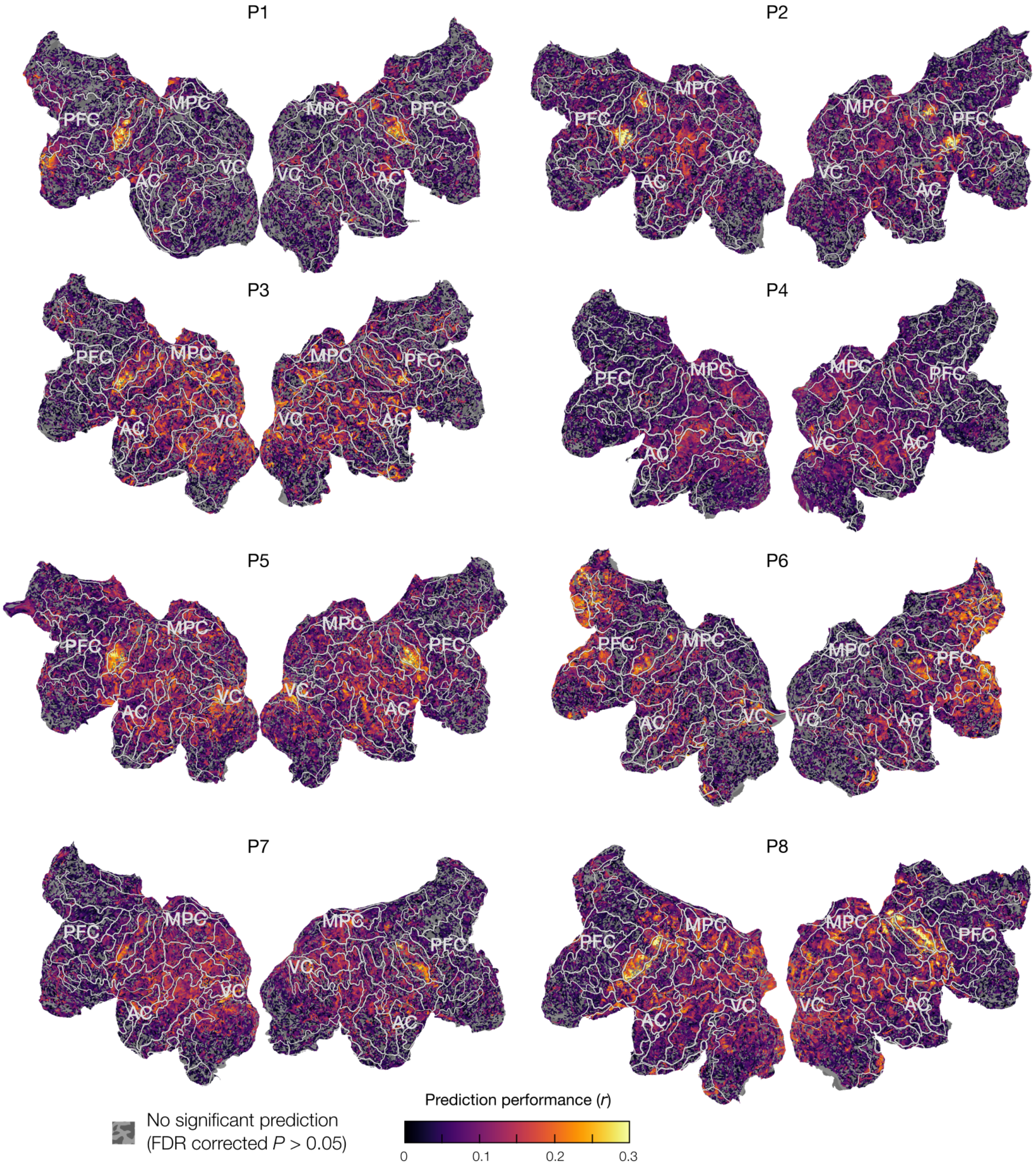


**Supplementary Fig. 3 | Cortical maps of Head Motion model prediction accuracy.** Flattened cortical surface maps illustrate the prediction accuracy within significantly predicted voxels (one-sided permutation test, $P < 0.05$, FDR corrected) for each participant. PFC: prefrontal cortex; MPC: medial parietal cortex; AC: auditory cortex; VC: visual cortex.

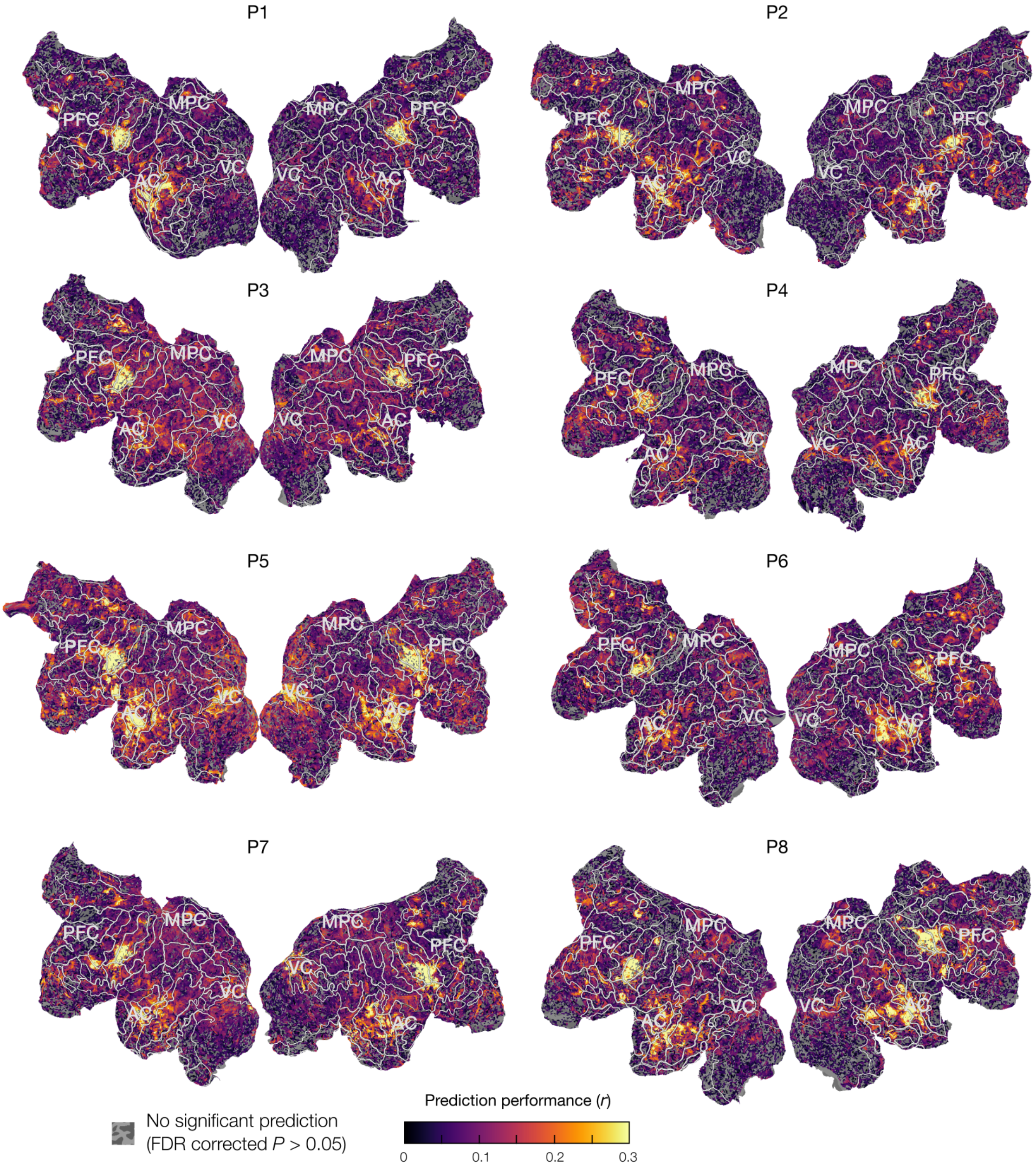


**Supplementary Fig. 4 | Cortical maps of prediction accuracy of random normal embeddings.** Flattened cortical surface maps illustrate the prediction accuracy within significantly predicted voxels (one-sided permutation test, $P < 0.05$, FDR corrected) for each participant. PFC: prefrontal cortex; MPC: medial parietal cortex; AC: auditory cortex; VC: visual cortex.

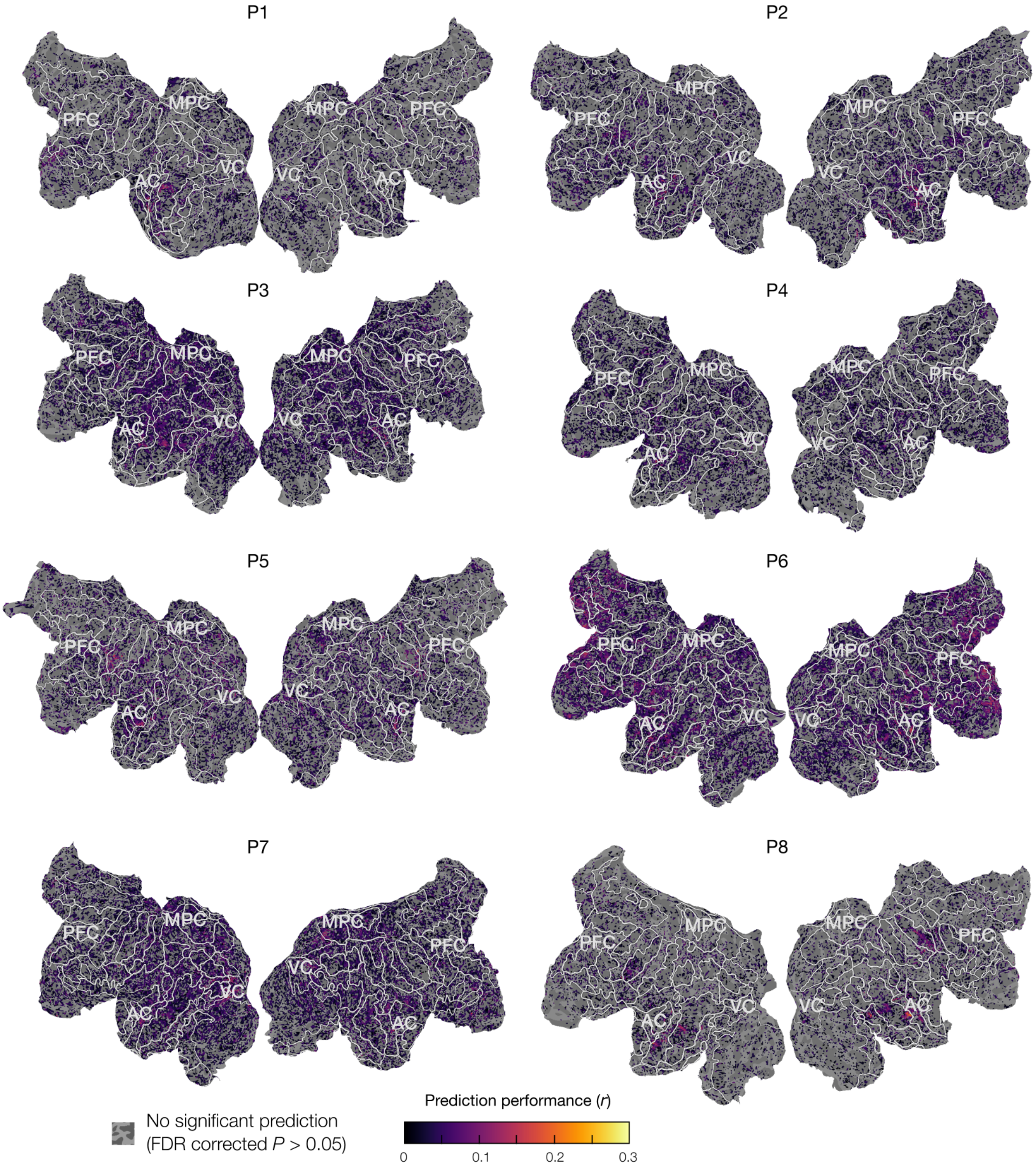


**Supplementary Fig. 5 | Cortical maps of prediction accuracy of additional low-level features.** Flattened cortical surface maps illustrate the prediction accuracy within significantly predicted voxels (one-sided permutation test, $P < 0.05$, FDR corrected) for each participant. PFC: prefrontal cortex; MPC: medial parietal cortex; AC: auditory cortex; VC: visual cortex.

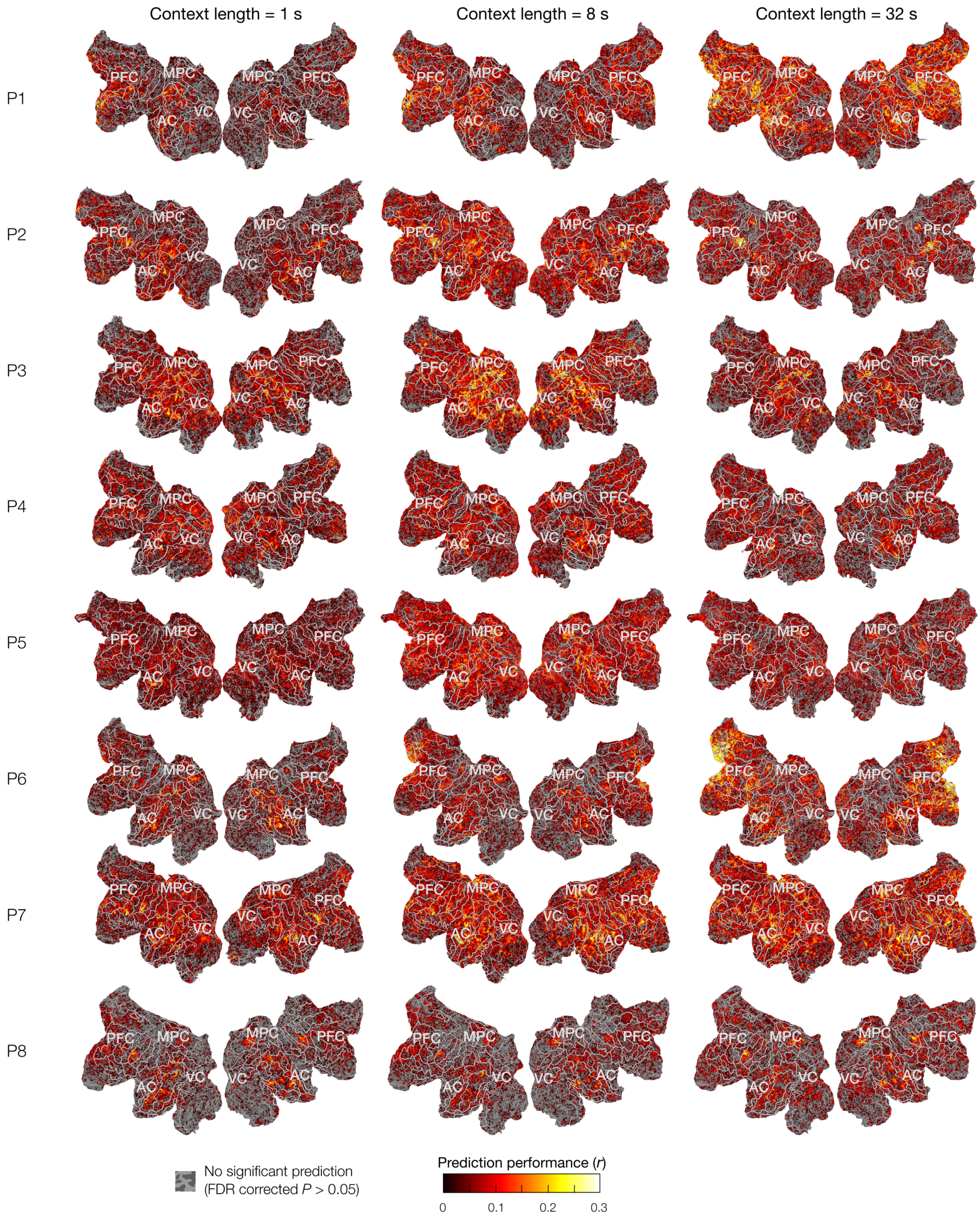


**Supplementary Fig. 6 | Cortical maps of Separate Linguistic model prediction accuracy for each participant.** Flattened cortical surface maps illustrate the prediction accuracy within significantly predicted voxels (one-sided permutation test, $P < 0.05$, FDR corrected) for each participant under three conditions: context lengths of 1, 8, and 32 s, using model layer 18. PFC: prefrontal cortex; MPC: medial parietal cortex; AC: auditory cortex; VC: visual cortex.

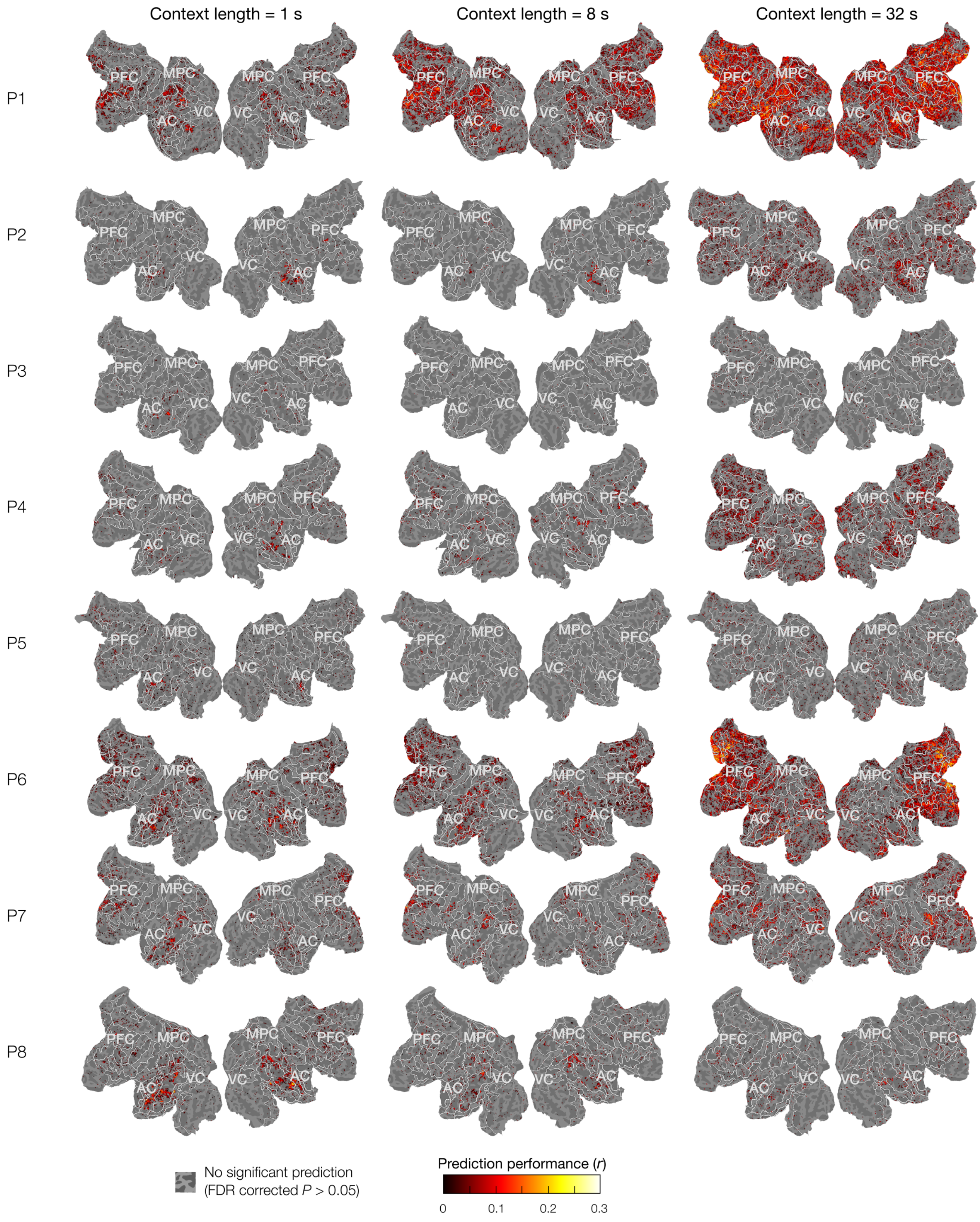


**Supplementary Fig. 7 | Cortical maps of cross-modality prediction accuracy for each participant.** Flattened cortical surface maps illustrate the prediction accuracy within significantly predicted voxels (one-sided permutation test, $P < 0.05$, FDR corrected) for each participant under three conditions: context lengths of 1, 8, and 32 s, using model layer 18. PFC: prefrontal cortex; MPC: medial parietal cortex; AC: auditory cortex; VC: visual cortex.

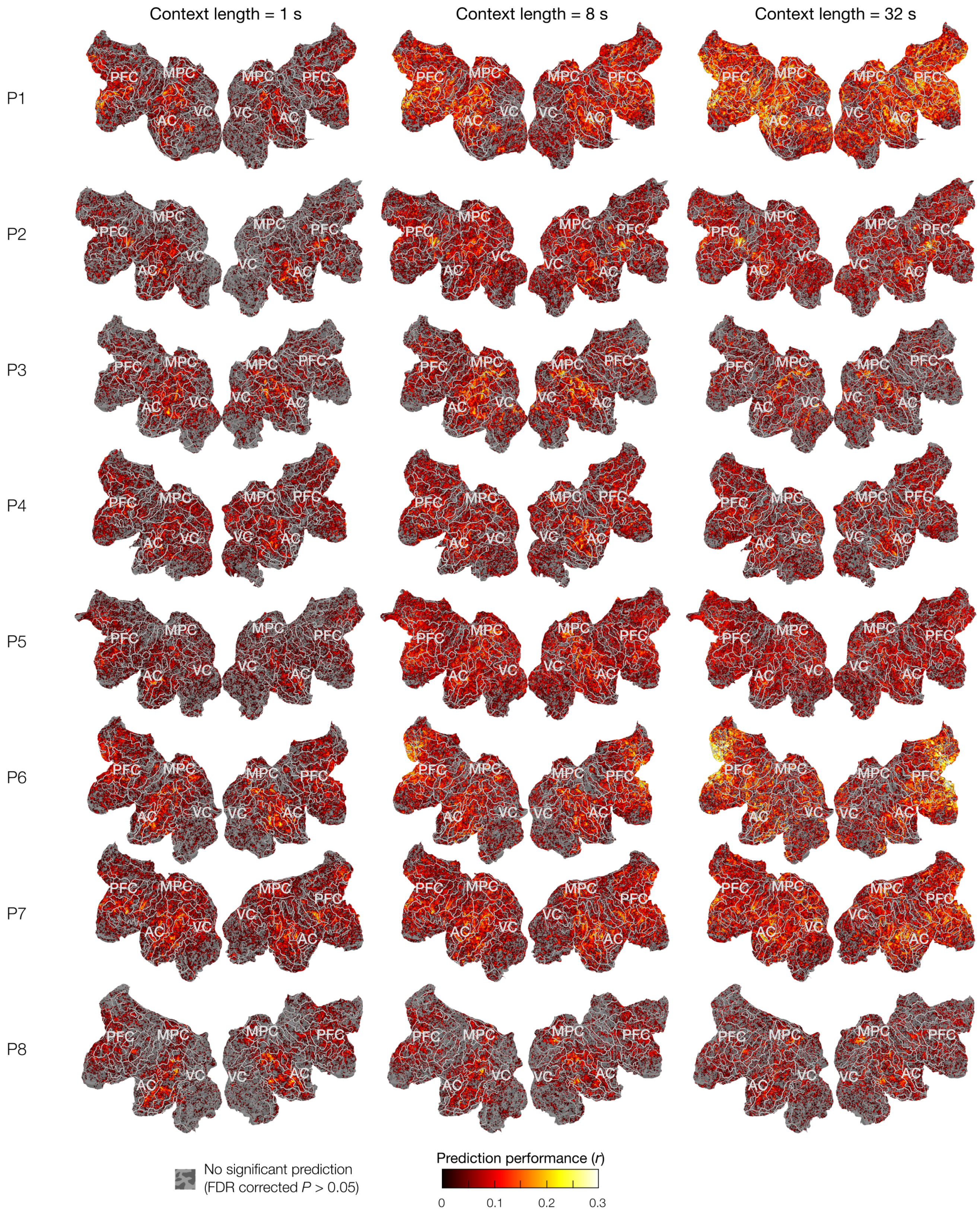


**Supplementary Fig. 8 | Cortical maps of Unified Linguistic model prediction accuracy for each participant.** Flattened cortical surface maps illustrate the prediction accuracy within significantly predicted voxels (one-sided permutation test, $P < 0.05$, FDR corrected) for each participant three conditions: context lengths of 1, 8, and 32 s, using model layer 18. PFC: prefrontal cortex; MPC: medial parietal cortex; AC: auditory cortex; VC: visual cortex.

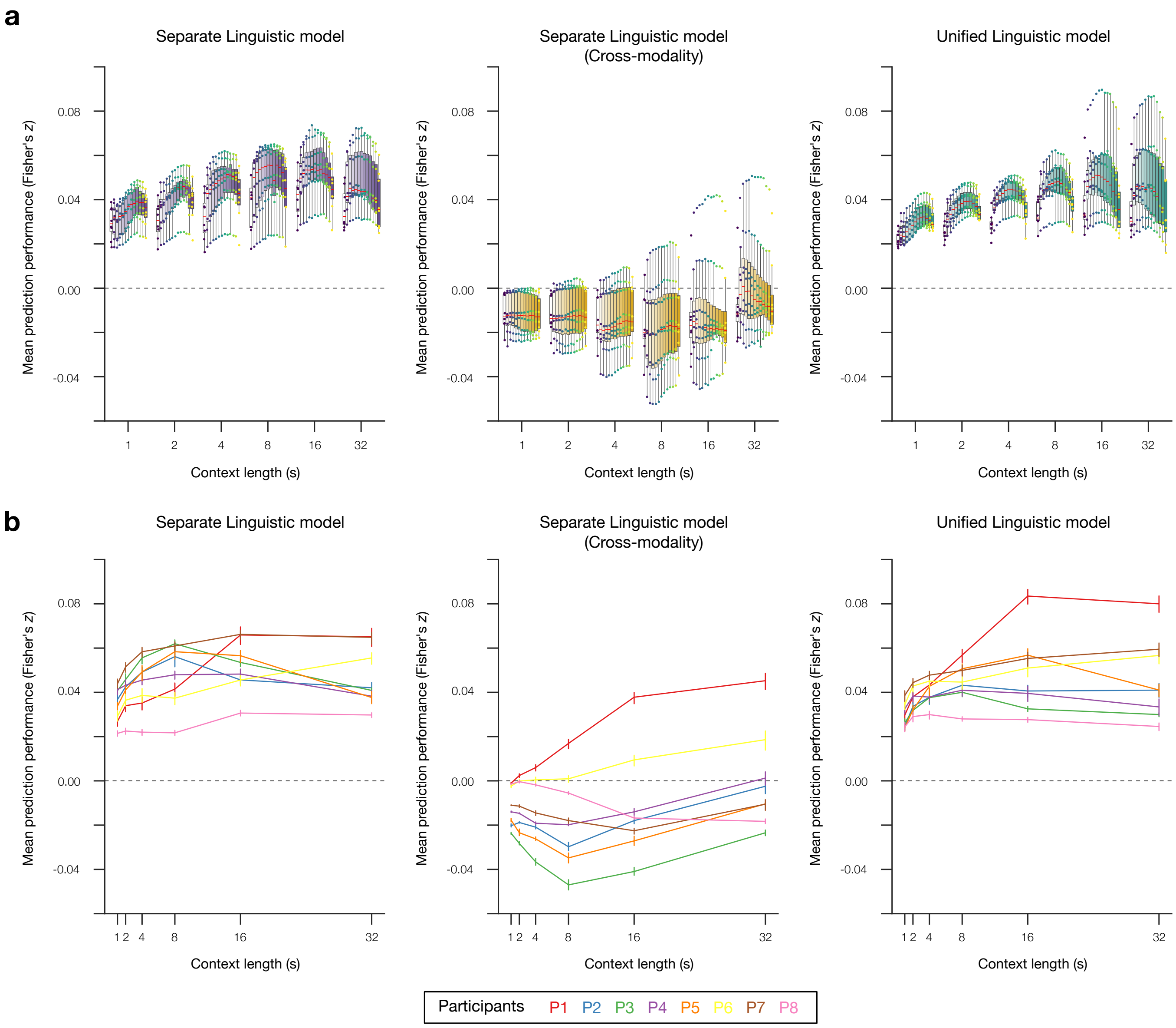


**Supplementary Fig. 9 | Prediction performance of linguistic models across context lengths and layers. a**, Box plots of the mean prediction performance across voxels for each context length and model layer (0, 3, …, 36, from left to right). Individual participants are represented by dots, with colors indicating model layers. The central red line within each box plot represents the median, while the box boundaries show the upper and lower quartiles. Whiskers extend to 1.5 times the interquartile range. **b**, Line plots of individual mean prediction performance across voxels, averaged across layers. Error bars indicate the standard deviation across layers.

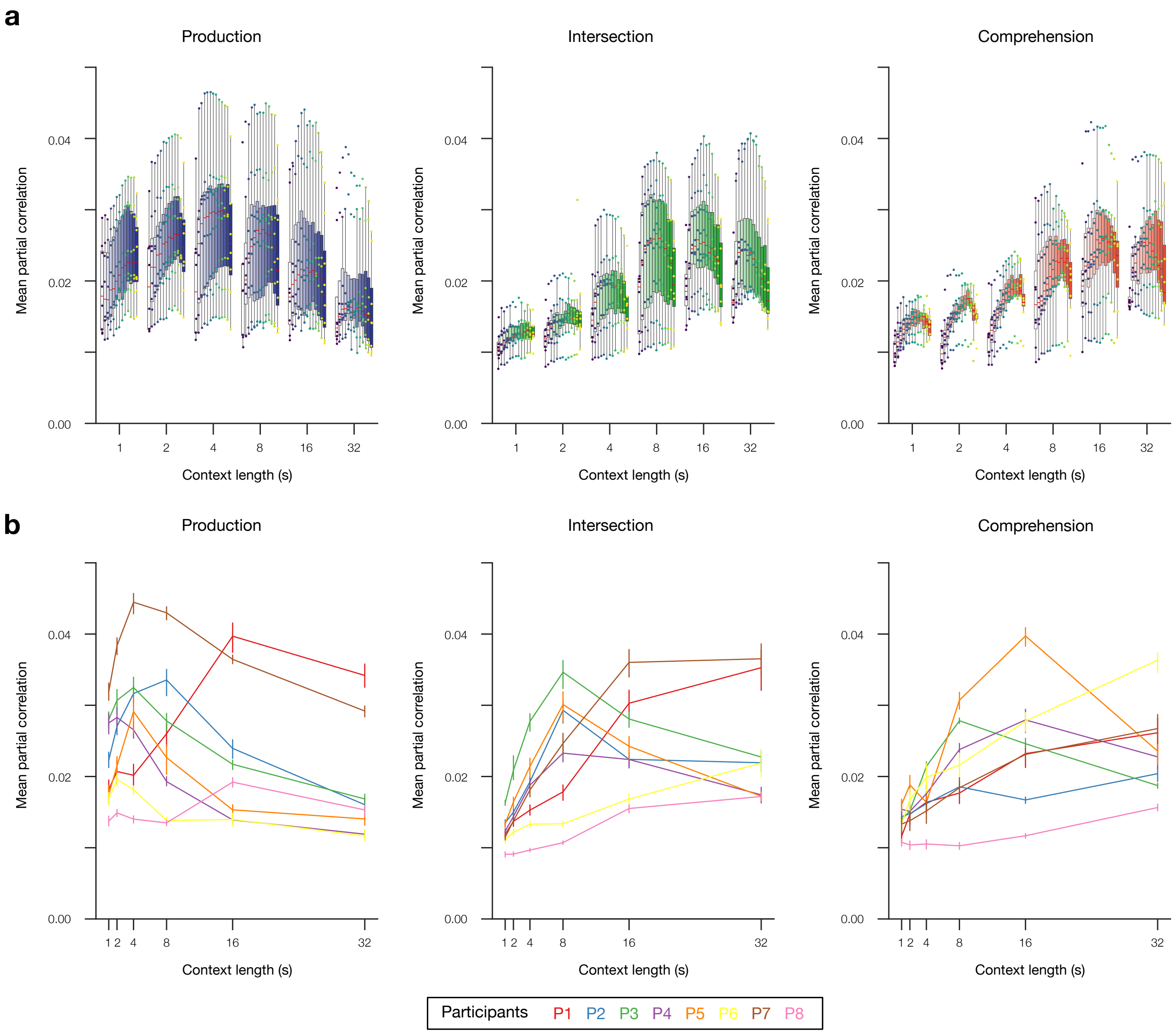


**Supplementary Fig. 10 | Variance explained by production, comprehension, and their intersection. a**, Box plots of the mean variance explained across voxels for each context length and model layer (0, 3, …, 36, from left to right). Individual participants are represented by dots, with colors indicating model layers. The central red line within each box plot represents the median, while the box boundaries show the upper and lower quartiles. Whiskers extend to 1.5 times the interquartile range. **b**, Line plots of the individual mean variance explained across voxels, averaged across layers. Error bars indicate the standard deviation across layers.

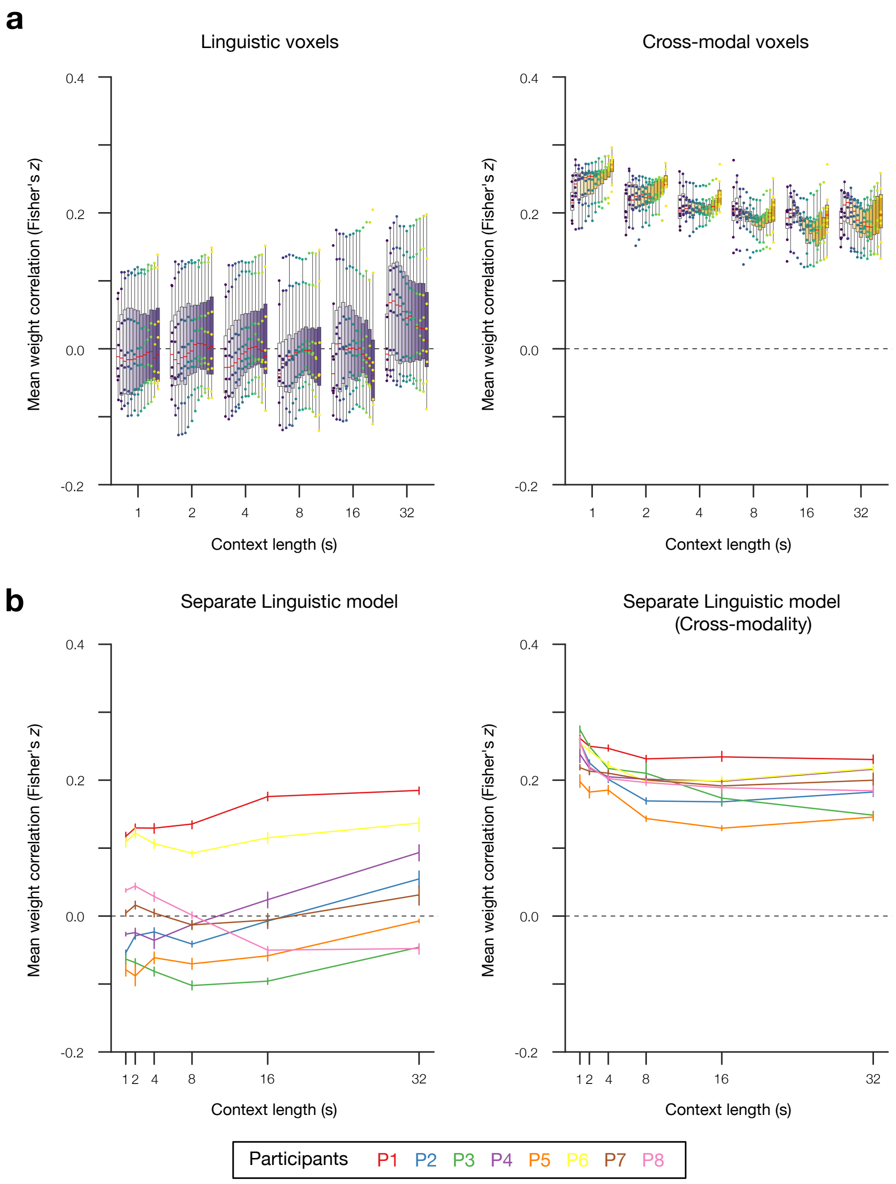


**Supplementary Fig. 11 | Weight correlation averaged across linguistic and cross-modal voxels. a**, Box plots of the mean weight correlation across voxels for each context length and model layer (0, 3, …, 36, from left to right). Individual participants are represented by dots, with colors indicating model layers. The central red line within each box plot represents the median, while the box boundaries show the upper and lower quartiles. Whiskers extend to 1.5 times the interquartile range. **b**, Line plots of the individual mean weight correlation across voxels, averaged across layers. Error bars indicate the standard deviation across layers.

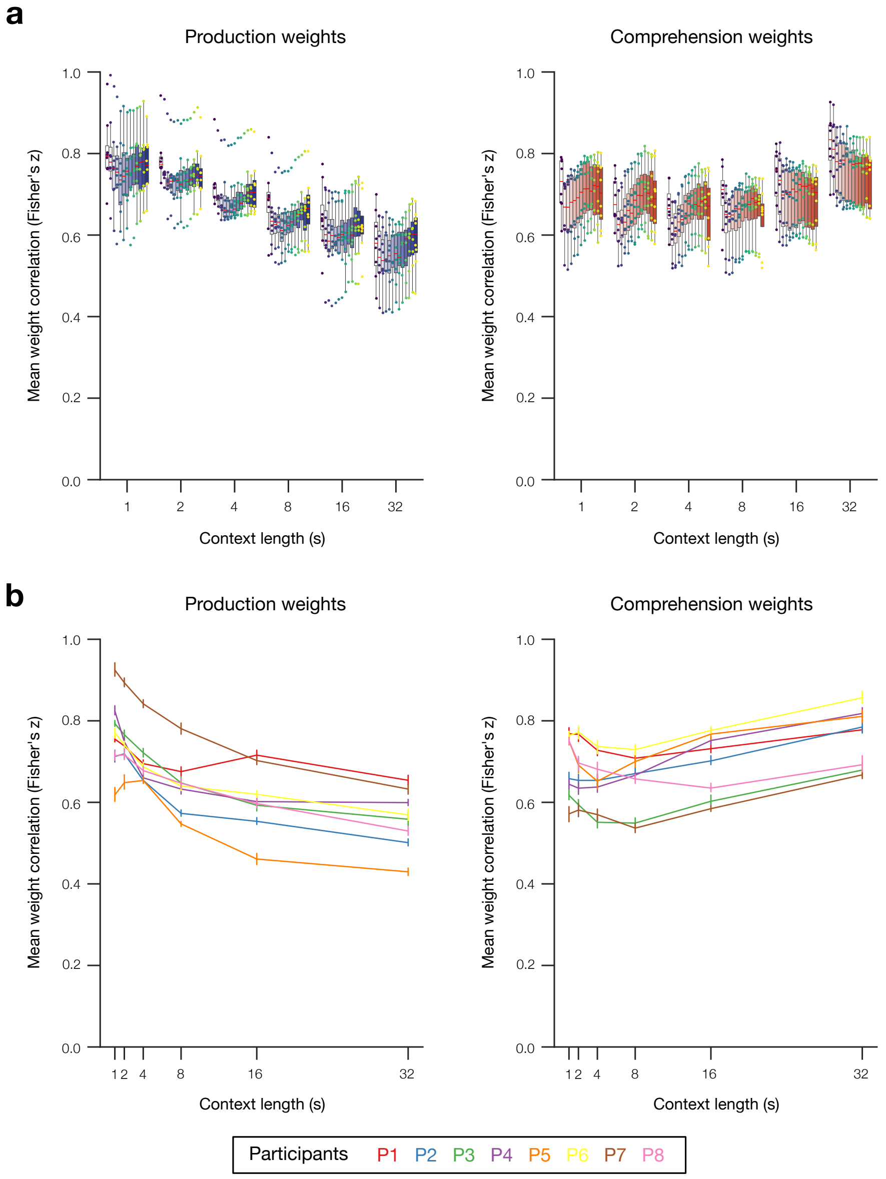


**Supplementary Fig. 12 | Weight correlation between the Unified and Separate Linguistic model. a**, Box plots of the mean weight correlation across voxels for each context length and model layer (0, 3, …, 36, from left to right). Individual participants are represented by dots, with colors indicating model layers. The central red line within each box plot represents the median, while the box boundaries show the upper and lower quartiles. Whiskers extend to 1.5 times the interquartile range. **b**, Line plots of the individual mean weight correlation across voxels, averaged across layers. Error bars indicate the standard deviation across layers.

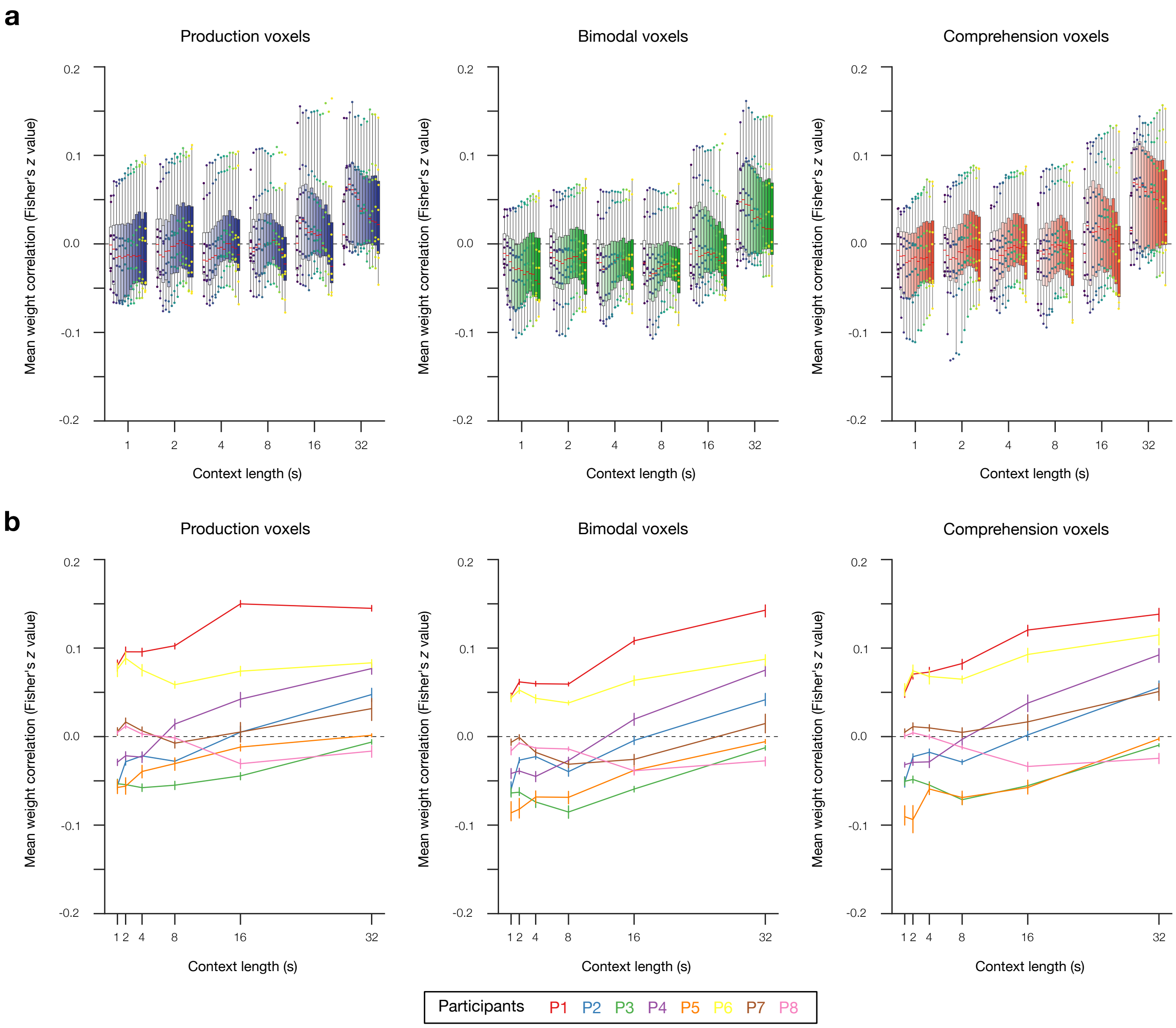


**Supplementary Fig. 13 | Weight correlation averaged across production, comprehension, and bimodal voxels. a**, Box plots of the mean weight correlation across voxels for each context length and model layer (0, 3, …, 36, from left to right). Individual participants are represented by dots, with colors indicating model layers. The central red line within each box plot represents the median, while the box boundaries show the upper and lower quartiles. Whiskers extend to 1.5 times the interquartile range. **b**, Line plots of the individual mean weight correlation across voxels, averaged across layers. Error bars indicate the standard deviation across layers.

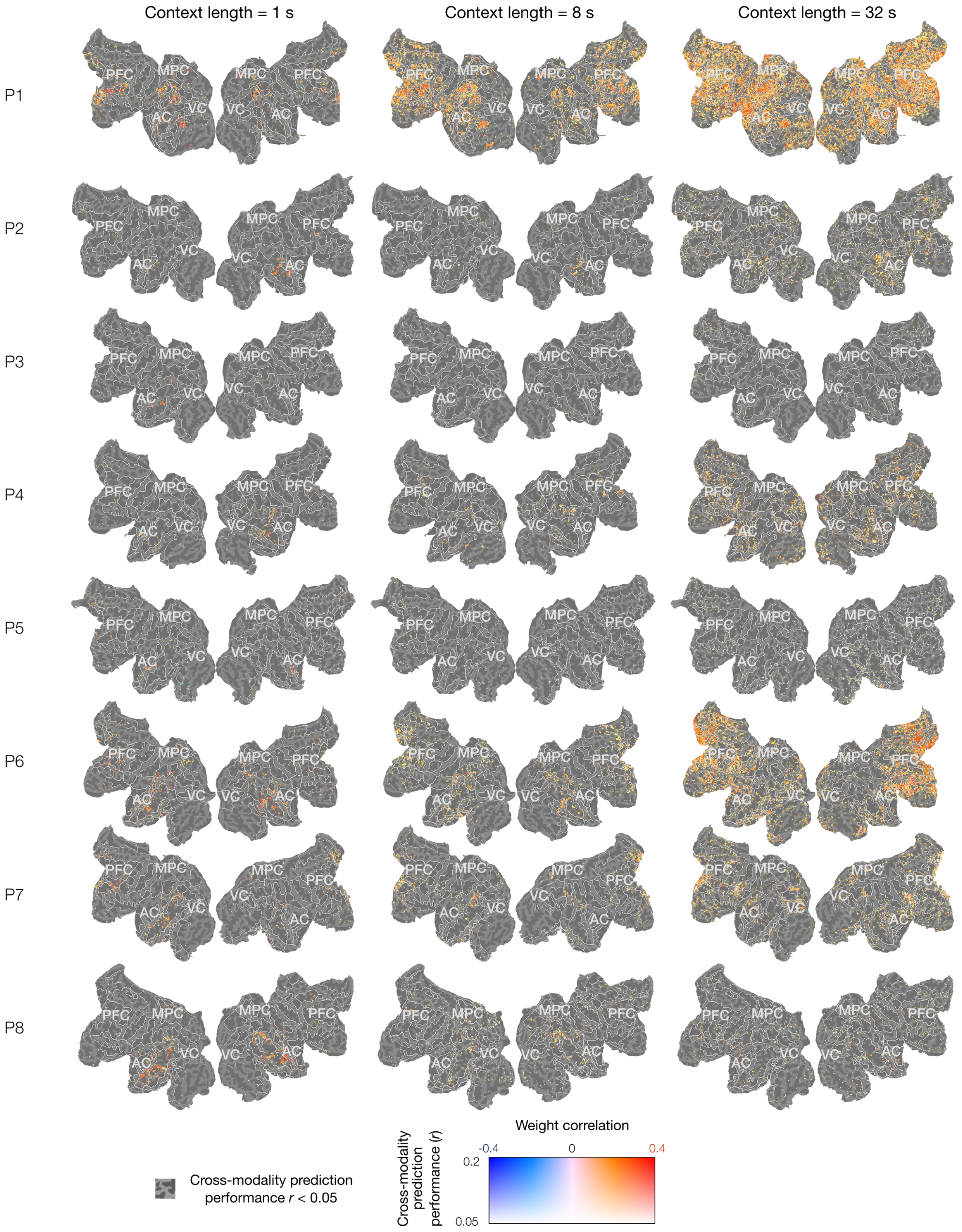


**Supplementary Fig. 14 | Cortical maps of weight correlation across cross-modal voxels.** Flattened cortical surface maps for each participant illustrate the weight correlation in cross-modal voxels—defined as voxels with prediction performance (Pearson's correlation coefficient) greater than 0.05 in both same-modality and cross-modality conditions—under three conditions: context lengths of 1, 8, and 32 s, using model layer 18. PFC: prefrontal cortex; MPC: medial parietal cortex; AC: auditory cortex; VC: visual cortex.

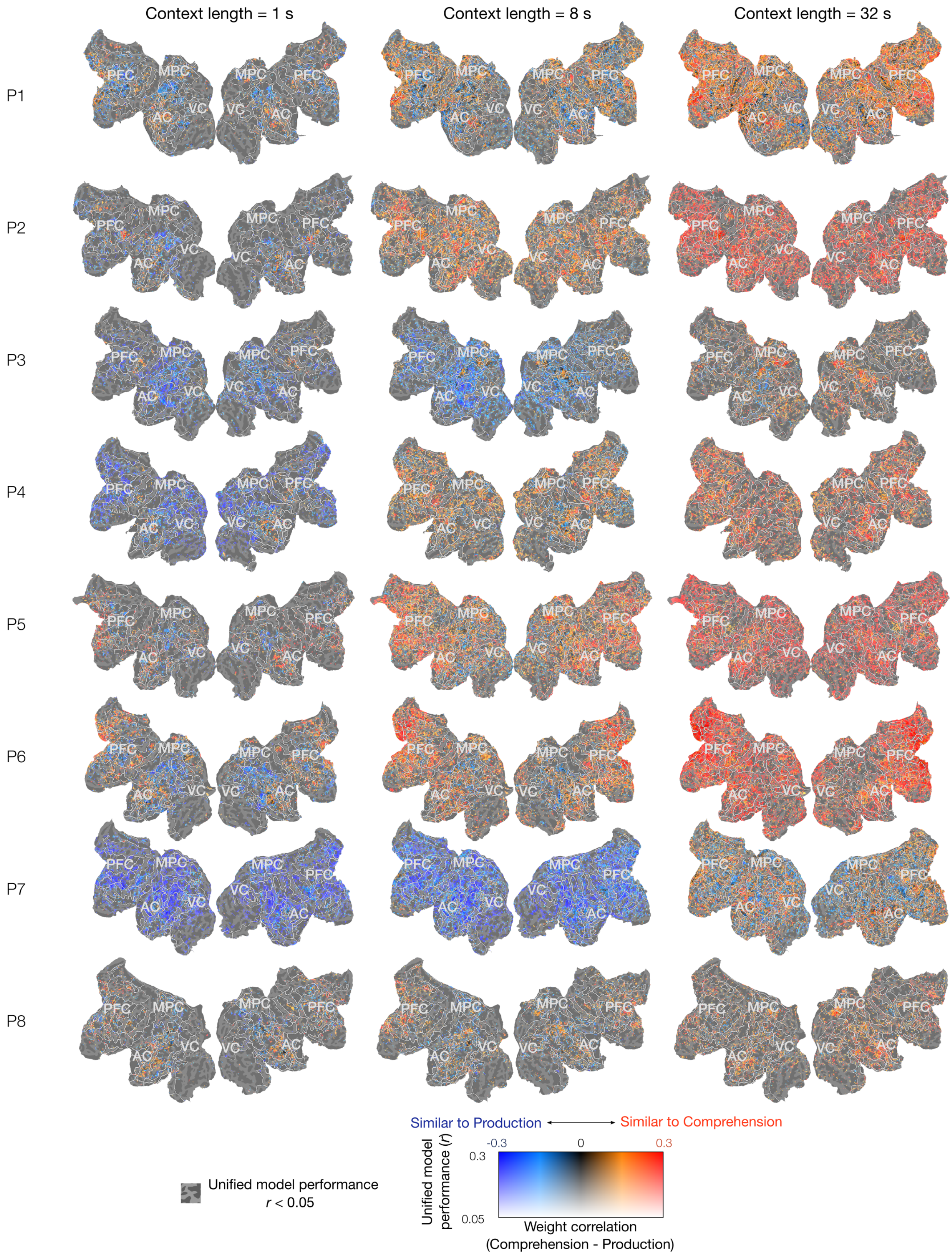


**Supplementary Fig. 15 | Cortical maps of weight correlation differences between the Unified and Separate Linguistic model.** Flattened cortical surface maps illustrate the weight correlation differences for each participant under three conditions: context lengths of 1, 8, and 32 s, using model layer 18. Only voxels demonstrating reliable prediction performance (Pearson's correlation coefficient $R > 0.05$) in the Unified Linguistic model are shown. PFC: prefrontal cortex; MPC: medial parietal cortex; AC: auditory cortex; VC: visual cortex.

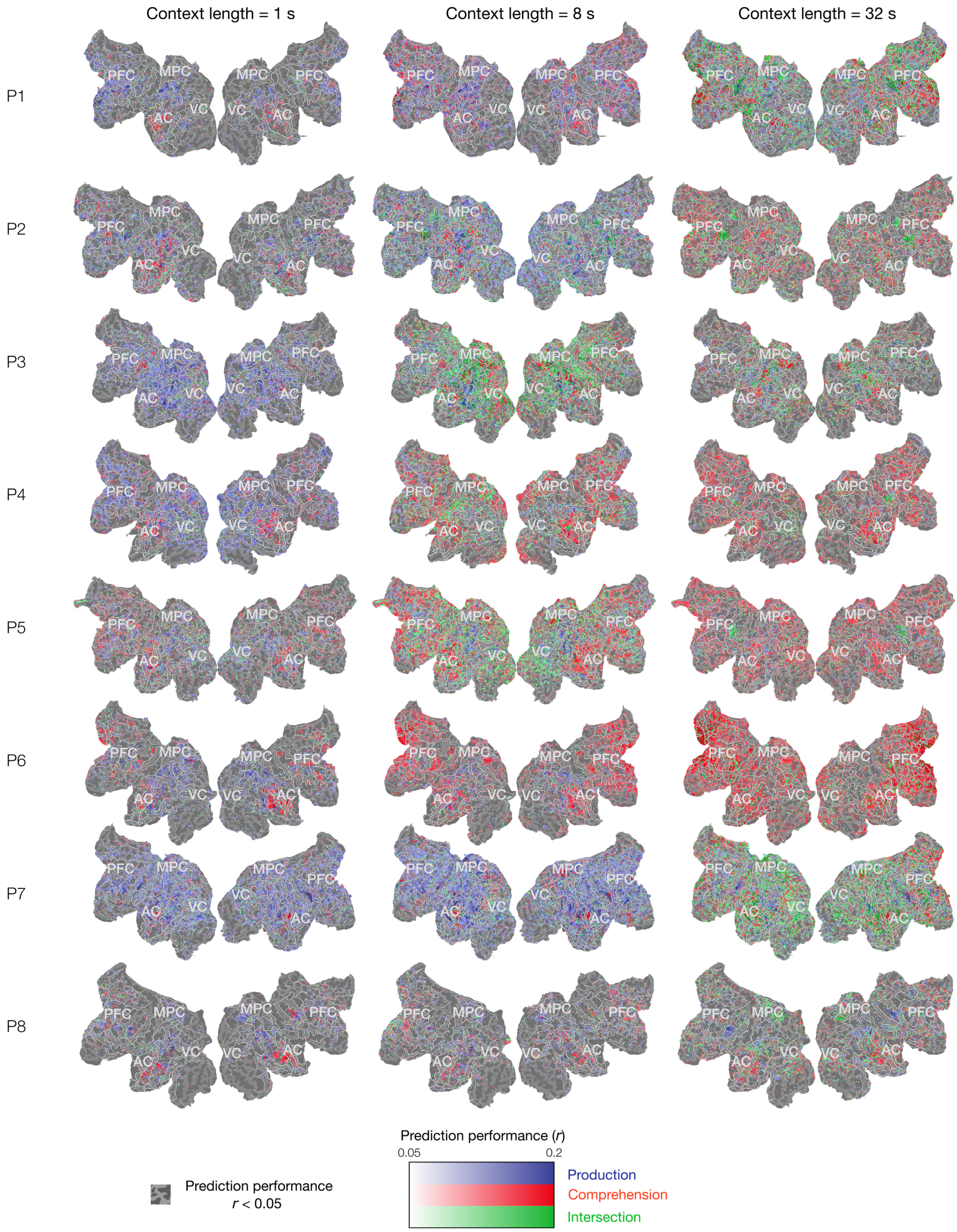


**Supplementary Fig. 16 | Cortical maps of the best variance partition.** Flattened cortical surface maps illustrate the best variance partition for each participant under three conditions: context lengths of 1, 8, and 32 s, using model layer 18. Only voxels demonstrating reliable prediction performance (Pearson's correlation coefficient $R > 0.05$) in the Separate Linguistic model are shown. PFC: prefrontal cortex; MPC: medial parietal cortex; AC: auditory cortex; VC: visual cortex.

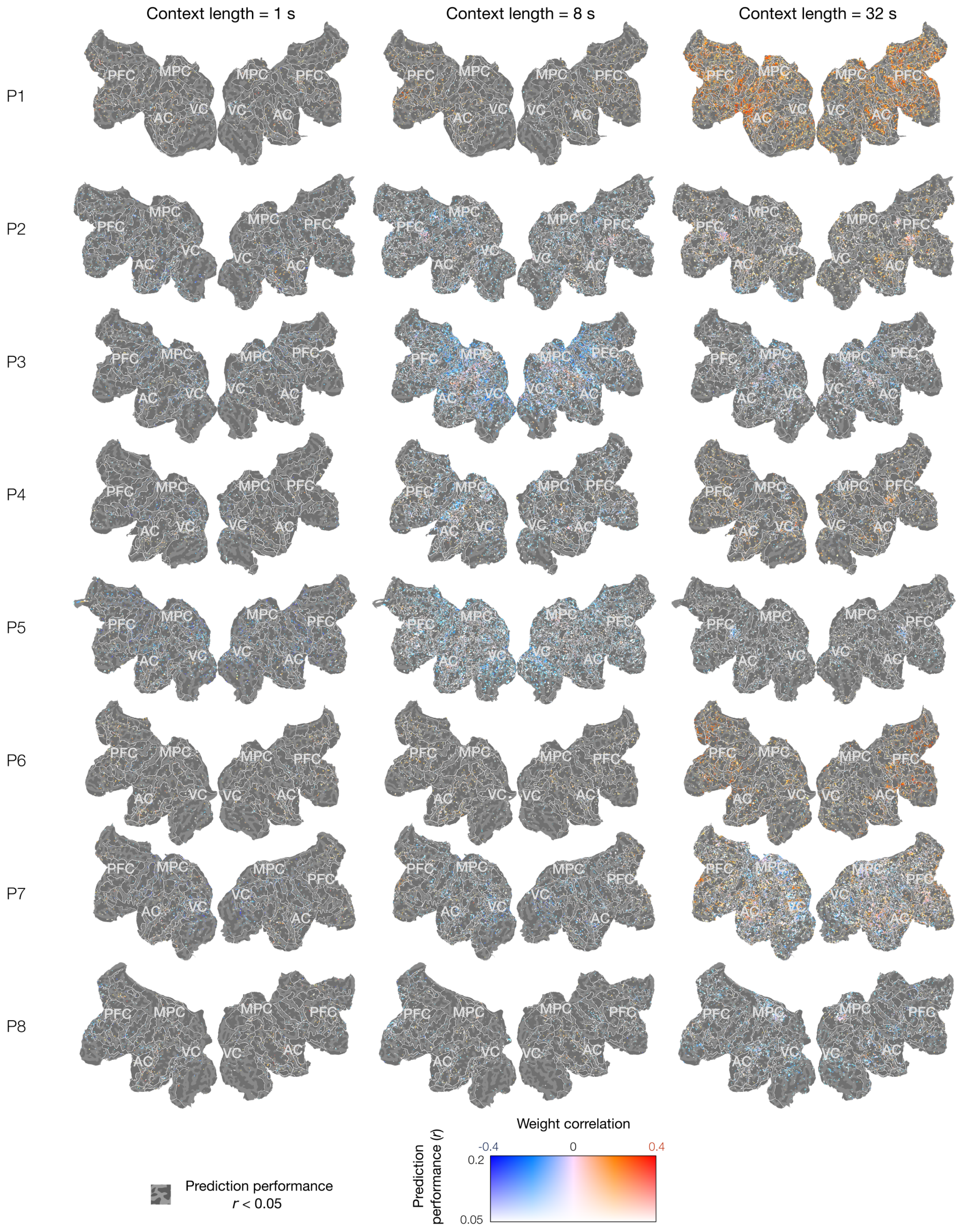


**Supplementary Fig. 17 | Cortical maps of weight correlation across bimodal voxels.** Flattened cortical surface maps illustrate the weight correlation in bimodal voxels for each participant under three conditions: context lengths of 1, 8, and 32 s, using model layer 18. Only voxels demonstrating reliable prediction performance (Pearson's correlation coefficient $R > 0.05$) in the Separate Linguistic model are shown. PFC: prefrontal cortex; MPC: medial parietal cortex; AC: auditory cortex; VC: visual cortex.

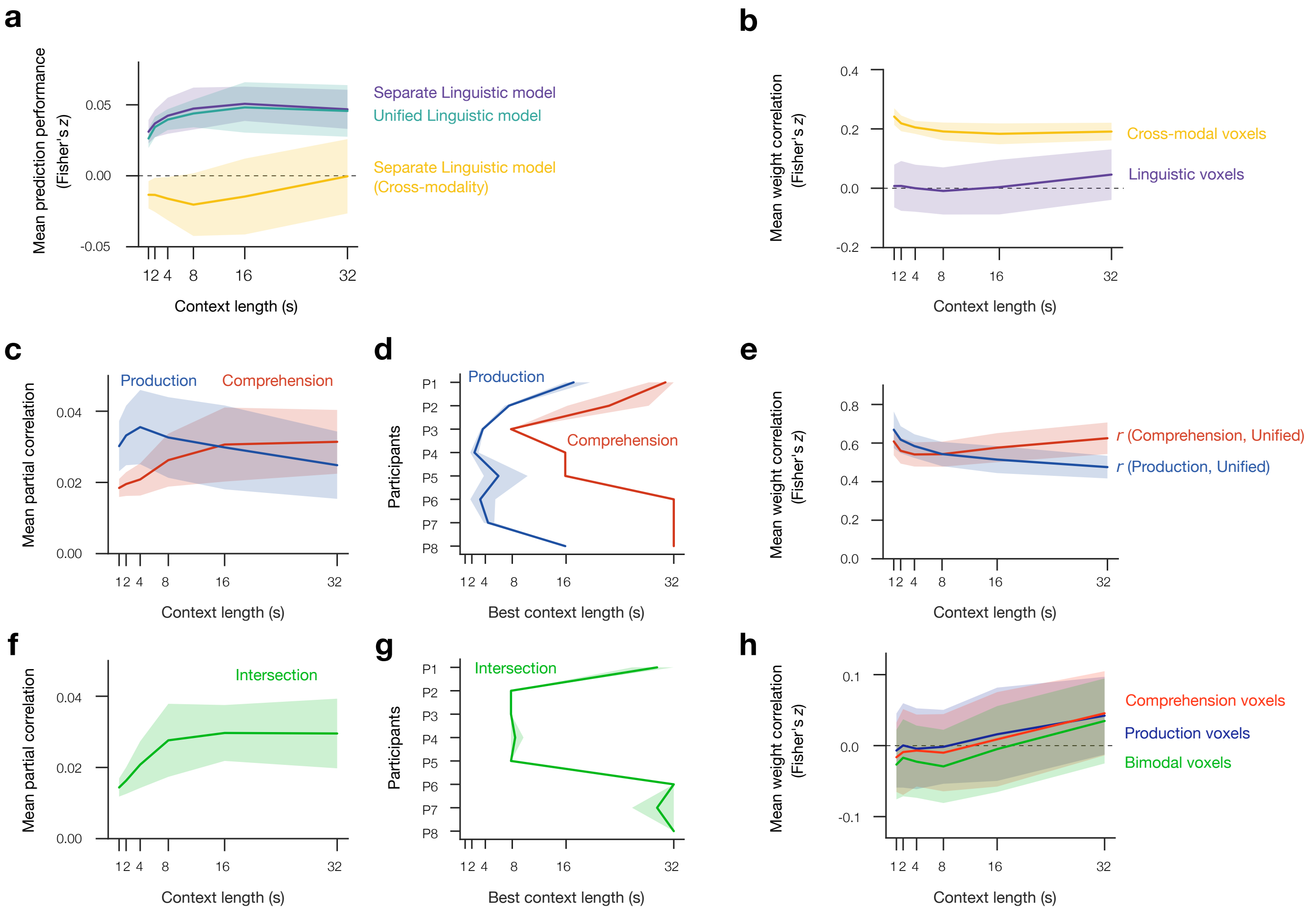


**Supplementary Fig. 18 | Replicated results using a base GPT model without instruction tuning. a**, Mean prediction performance (across voxels and layers) averaged across participants for each model. Cross-modality predictions showed significantly lower accuracy than the actual model (actual model: $t(7) = 6.88$, two-sided parametric test, $P = 2.4 \times 10^{-4}$, $\beta = 0.056$, 95% CI = [0.039, 0.072]). The Unified Linguistic model predictions showed significantly lower accuracy than the Separate Linguistic model (Separate Linguistic model: $t(1227) = 6.95$, two-sided parametric test, $P = 5.9 \times 10^{-12}$, $\beta = 2.9 \times 10^{-3}$, 95% CI = [$2.1 \times 10^{-3}$, $3.7 \times 10^{-3}$]). **b**, Mean weight correlation (across layers) across participants averaged for linguistic voxels and cross-modal voxels. Cross-modal voxels exhibited significantly higher weight correlation than linguistic voxels (cross-modal voxels: $t(7) = 10.88$, two-sided parametric test, $P = 1.2 \times 10^{-5}$, $\beta = 0.21$, 95% CI = [0.17, 0.25]). **c**, Mean unique variance (across voxels and layers) explained by production or comprehension, averaged across participants. **d**, Mean context lengths (across layers) that maximize the variance uniquely explained by production and comprehension for each participant. **e**, Mean weight correlation (across voxels and layers) between the Unified and Separate model weights for each modality, averaged across participants. **f**, Mean variance (across voxels and layers) uniquely explained by the intersection of production and comprehension, averaged across participants. **g**, Mean context lengths (across layers) that maximize the unique variance explained by the intersection for each participant. **h**, Mean weight correlation (across voxels and layers) of each partition, averaged across participants. Bimodal voxels exhibited significantly lower weight correlation than production voxels (production: $t(7) = 10.31$, two-sided parametric test, $P = 1.8 \times 10^{-5}$, $\beta = 0.030$, 95% CI = [0.024, 0.036]) and comprehension voxels (comprehension: $t(1229) = 16.49$, two-sided parametric test, $P < 2.2 \times 10^{-16}$, $\beta = 0.022$, 95% CI = [0.019, 0.024]). The shaded area show the standard deviation across participants.

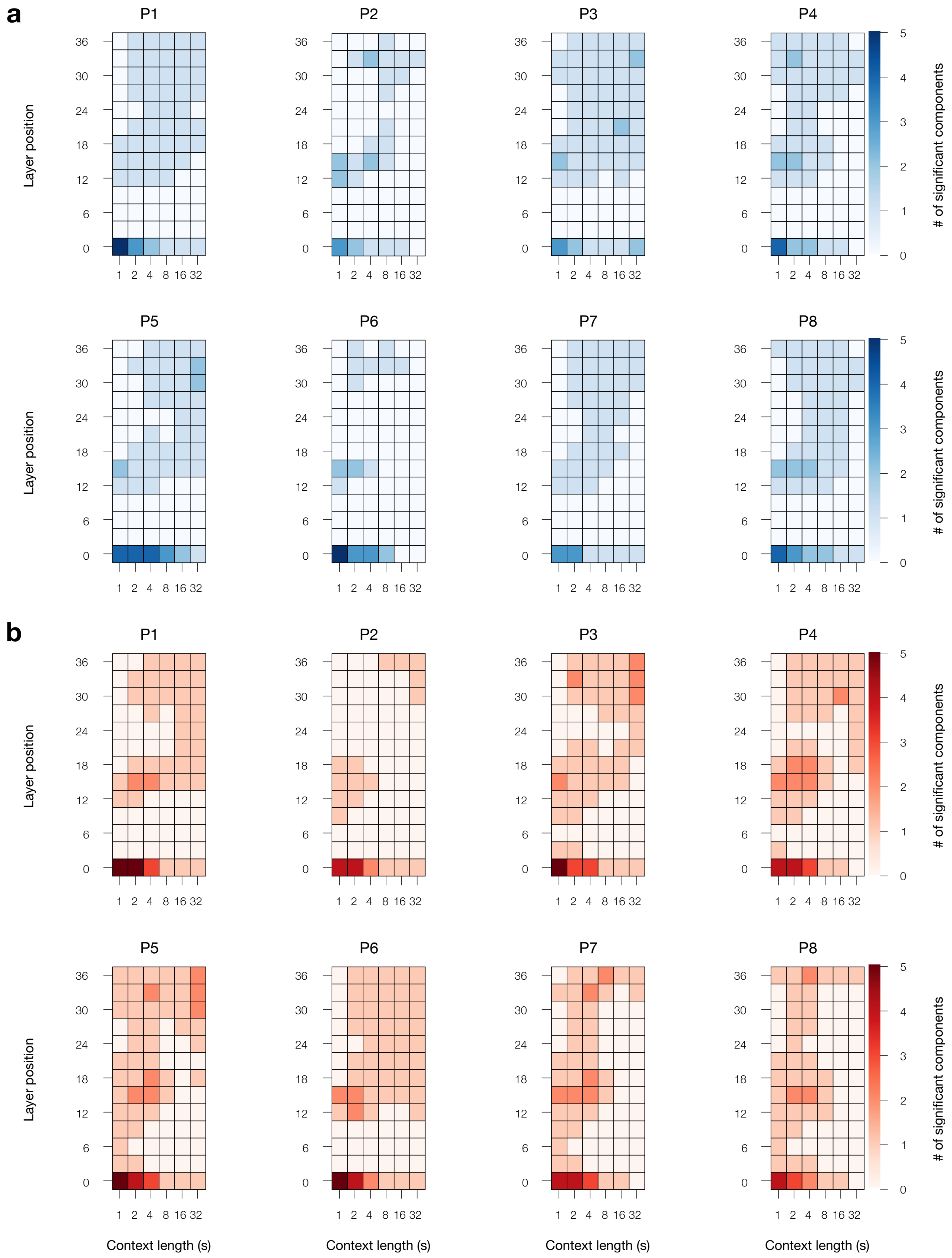


**Supplementary Fig. 19 | The number of significant principal components (PCs) identified for each participant. a**, Results from Production PCA. **b**, Results from Comprehension PCA.

## Supplementary Tables

### Supplementary Table 1 | Linear mixed-effects model statistics for prediction accuracy.

| **Separate Linguistic model** | ***t*** | ***df*** | ***P*** | ***β*** | ***95% CI*** |
|---|---|---|---|---|---|
| Context length | 4.41 | 7.0 | **3.10E-03** | 0.733 | [0.388, 1.079] |
| (Context length)^2 | -4.54 | 7.0 | **2.65E-03** | -0.434 | [-0.632, -0.235] |
| Layer | 11.36 | 597.0 | **< 2E-16** | 0.146 | [0.121, 0.171] |
| (Layer)^2 | -17.95 | 597.0 | **< 2E-16** | -0.260 | [-0.288, -0.232] |
| Layer: Context length | -6.95 | 597.0 | **9.73E-12** | -0.089 | [-0.114, -0.064] |
| **Cross-modality** | ***t*** | ***df*** | ***P*** | ***β*** | ***95% CI*** |
| Context length | 4.01 | 7.0 | **5.16E-03** | 0.403 | [0.146, 0.661] |
| Layer | 3.91 | 597.0 | **1.02E-04** | 0.032 | [0.016, 0.048] |
| (Layer)^2 | -3.39 | 597.0 | **7.34E-04** | -0.032 | [-0.050, -0.013] |
| Layer: Context length | -2.75 | 597.0 | **6.19E-03** | -0.023 | [-0.039, -0.007] |
| **Unified Linguistic model** | ***t*** | ***df*** | ***P*** | ***β*** | ***95% CI*** |
| Layer | 7.45 | 597.0 | **3.41E-13** | 0.083 | [0.0611, 0.105] |
| (Layer)^2 | -22.79 | 597.0 | **< 2E-16** | -0.287 | [-0.312, -0.262] |
| Layer: Context_length | -9.45 | 597.0 | **< 2E-16** | -0.105 | [-0.127, -0.084] |
| **Production-only model** | ***t*** | ***df*** | ***P*** | ***β*** | ***95% CI*** |
| Context length | -3.87 | 7.0 | **6.13E-03** | -0.396 | [-0.615, -0.177] |
| Layer | 3.72 | 597.0 | **2.19E-04** | 0.049 | [0.023, 0.075] |
| (Layer)^2 | -8.82 | 597.0 | **< 2E-16** | -0.132 | [-0.162, -0.103] |
| Layer: Context_length | -5.27 | 597.0 | **1.87E-07** | -0.070 | [-0.096, -0.044] |
| **Comprehension-only model** | ***t*** | ***df*** | ***P*** | ***β*** | ***95% CI*** |
| Context length | 4.55 | 7.0 | **2.64E-03** | 1.019 | [0.554, 1.485] |
| (Context length)^2 | -2.42 | 7.0 | **0.046** | -0.406 | [-0.754, -0.058] |
| Layer | 8.33 | 598.0 | **5.51E-16** | 0.105 | [0.081, 0.130] |
| (Layer)^2 | -11.57 | 598.0 | **< 2E-16** | -0.165 | [-0.193, -0.137] |
| **Intersection** | ***t*** | ***df*** | ***P*** | ***β*** | ***95% CI*** |
| Context length | 6.18 | 7.0 | **4.53E-04** | 1.016 | [0.675, 1.358] |
| (Context length)^2 | -4.59 | 7.0 | **2.52E-03** | -0.486 | [-0.706, -0.266] |
| Layer | 0.99 | 597.0 | 0.321 | 0.014 | [-0.014, 0.042] |
| Layer^2 | -12.30 | 597.0 | **< 2E-16** | -0.199 | [-0.231, -0.167] |
| Layer: Context_length | -3.04 | 597.0 | **2.50E-03** | -0.044 | [-0.072, -0.016] |
| **Separate Linguistic model: Actual vs. Cross-modality** | ***t*** | ***df*** | ***P*** | ***β*** | ***95% CI*** |
| Model (Actual) | 7.05 | 7.0 | **2.01E-04** | 0.057 | [0.040, 0.074] |
| Context length | 2.55 | 7.4 | **0.036** | 4.91E-04 | [9.31E-05, 8.89E-04] |
| Layer | 6.73 | 1222.0 | **2.66E-11** | 1.84E-04 | [1.30E-04, 2.36E-04] |
| Layer: Context_length | -3.68 | 1222.0 | **2.40E-04** | -6.66E-06 | [-1.02E-05, -3.12E-06] |
| **Separate Linguistic model vs. Unified Linguistic model** | ***t*** | ***df*** | ***P*** | ***β*** | ***95% CI*** |
| Model (Separate) | 9.03 | 1227.0 | **< 2E-16** | 3.39E-03 | [2.66E-03, 4.13E-03] |
| Context length | 10.64 | 9.5 | **1.36E-06** | 2.09E-03 | [1.69E-03, 2.49E-03] |
| (Context length)^2 | -22.44 | 10.6 | **< 2E-16** | -4.69E-05 | [-5.10E-05, -4.28E-05] |
| Layer | 19.70 | 1227.0 | **< 2E-16** | 1.28E-03 | [1.15E-03, 1.40E-03] |
| (Layer)^2 | -17.06 | 1227.0 | **< 2E-16** | -2.87E-05 | [-3.20E-05, -2.54E-05] |
| Layer: Context_length | -6.85 | 1227.0 | **1.14E-11** | -1.06E-05 | [-1.36E-05, -7.56E-06] |

The ‘Separate Linguistic model: Actual vs. Cross-modality’ comparison includes a random intercept and a random slope (1 + type + context | participant). The ‘Separate Linguistic model vs. Unified Linguistic model’ comparison includes both a random intercept and a random slope (1 + context | participant). Other models incorporate random intercepts, random slopes, and a quadratic term (1 + context + I(context^2) | participant).

## Supplementary Table 2 | Linear mixed-effects model statistics for weight correlation.

| **Linguistic voxels** | ***t*** | ***df*** | ***P*** | ***β*** | ***95% CI*** |
|---|---|---|---|---|---|
| Context length | 5.02 | 7.0 | **1.5E-03** | 0.389 | [0.204, 0.573] |
| Layer | 4.64 | 597.0 | **4.3E-06** | 0.037 | [0.021, 0.052] |
| (Layer)^2 | -4.99 | 597.0 | **7.8E-07** | -0.045 | [-0.062, -0.027] |
| Layer: Context_length | -5.42 | 597.0 | **8.8E-08** | -0.043 | [-0.059, -0.028] |
| **Cross-modal voxels** | ***t*** | ***df*** | ***P*** | ***β*** | ***95% CI*** |
| Context length | -5.89 | 7.0 | **6.1E-04** | -1.015 | [-1.373, -0.657] |
| (Context length)^2 | 7.18 | 7.0 | **1.8E-04** | 0.516 | [0.367, 0.666] |
| Layer | 3.06 | 597.0 | **2.3E-03** | 0.049 | [0.018, 0.080] |
| (Layer)^2 | 5.86 | 597.0 | **7.7E-09** | 0.105 | [0.070, 0.140] |
| Layer: Context_length | -7.45 | 597.0 | **3.4E-13** | -0.118 | [-0.149, -0.087] |
| **Production voxels** | ***t*** | ***df*** | ***P*** | ***β*** | ***95% CI*** |
| Context length | 3.94 | 7.0 | **5.6E-03** | 0.272 | [0.126, 0.417] |
| Layer | 2.42 | 597.0 | **0.016** | 0.019 | [0.004, 0.035] |
| (Layer)^2 | -6.90 | 597.0 | **1.3E-11** | -0.062 | [-0.080, -0.045] |
| Layer: Context_length | -8.07 | 597.0 | **3.8E-15** | -0.065 | [-0.080, -0.049] |
| **Comprehension voxels** | ***t*** | ***df*** | ***P*** | ***β*** | ***95% CI*** |
| Context length | 4.89 | 7.0 | **1.8E-03** | 0.385 | [0.212, 0.557] |
| Layer | 8.30 | 597.0 | **7.2E-16** | 0.068 | [0.052, 0.085] |
| (Layer)^2 | -5.73 | 597.0 | **1.6E-08** | -0.053 | [-0.072, -0.035] |
| Layer: Context_length | -5.24 | 597.0 | **2.2E-07** | -0.043 | [-0.060, -0.027] |
| **Bimodal voxels** | ***t*** | ***df*** | ***P*** | ***β*** | ***95% CI*** |
| Context length | 5.93 | 7.0 | **5.8E-04** | 0.494 | [0.272, 0.715] |
| Layer | 1.10 | 598.0 | 0.274 | 0.010 | [0.008, 0.027] |
| Layer: Context_length | -2.71 | 598.0 | **6.9E-03** | -0.024 | [-0.042, -0.007] |
| **Unified vs. Production** | ***t*** | ***df*** | ***P*** | ***β*** | ***95% CI*** |
| Context length | -8.59 | 7.0 | **5.8E-05** | -1.013 | [-1.257, -0.768] |
| (Context length)^2 | 5.59 | 7.0 | **8.2E-04** | 0.317 | [0.199, 0.435] |
| Layer | 2.97 | 597.0 | **3.1E-03** | 0.029 | [0.010, 0.049] |
| (Layer)^2 | 12.71 | 597.0 | **< 2E-16** | 0.142 | [0.120, 0.164] |
| Layer: Context_length | 4.76 | 597.0 | **2.4E-06** | 0.047 | [0.028, 0.067] |
| **Unified vs. Comprehension** | ***t*** | ***df*** | ***P*** | ***β*** | ***95% CI*** |
| Context length | 0.70 | 7.0 | 0.505 | 0.119 | [-0.233, 0.472] |
| (Context length)^2 | 3.30 | 7.0 | **0.013** | 0.229 | [0.085, 0.372] |
| Layer | 2.85 | 597.0 | **4.6E-03** | 0.040 | [0.012, 0.067] |
| (Layer)^2 | 2.20 | 597.0 | **0.028** | 0.035 | [0.004, 0.066] |
| Layer: Context_length | -11.48 | 597.0 | **< 2E-16** | -0.161 | [-0.188, -0.133] |

All models incorporate random intercepts, random slopes, and a quadratic term (1 + context + I(context^2) | participant).

## Supplementary Table 3 | Linear mixed-effects model statistics for weight correlation comparisons.

| **Linguistic voxels vs. Cross-modal voxels** | ***t*** | ***df*** | ***P*** | ***β*** | ***95% CI*** |
|---|---|---|---|---|---|
| Voxel type (Cross-modal) | 11.35 | 7.0 | **9.2E-06** | 0.203 | [0.166, 0.240] |
| Context length | -7.74 | 1228.0 | **2.1E-14** | -2.6E-03 | [-3.3E-03, -2.0E-03] |
| (Context length)^2 | 10.93 | 1228.0 | **< 2E-16** | 1.0E-04 | [8.3E-05, 1.2E-04] |
| Layer | 4.92 | 1228.0 | **9.7E-07** | 5.1E-04 | [3.1E-04, 7.1E-04] |
| Layer: Context_length | -4.38 | 1228.0 | **1.3E-05** | -3.0E-05 | [-4.4E-05, -1.7E-05] |
| **Bimodal voxels vs. Production voxels** | ***t*** | ***df*** | ***P*** | ***β*** | ***95% CI*** |
| Voxel type (Production) | 11.99 | 8.4 | **1.4E-06** | 0.038 | [0.032, 0.045] |
| Voxel type (Production):Context length | -8.58 | 1221.0 | **< 2E-16** | -7.7E-04 | [-9.4E-04, -5.9E-04] |
| Context length | 4.07 | 7.2 | **4.4E-03** | 2.7E-03 | [1.3E-03, 4.1E-03] |
| Layer | 5.83 | 1221.0 | **7.0E-09** | 3.5E-04 | [2.3E-04, 4.7E-04] |
| Layer: Context_length | -6.26 | 1221.0 | **5.4E-10** | -2.5E-05 | [-3.3E-05, -1.7E-05] |
| **Bimodal voxels vs. Comprehension voxels** | ***t*** | ***df*** | ***P*** | ***β*** | ***95% CI*** |
| Voxel type (Comprehension) | -21.00 | 1227.0 | **< 2e-16** | 0.021 | [0.019, 0.023] |
| Context length | 1.65 | 8.2 | 0.137 | 1.2E-03 | [-3.0E-04, 2.7E-03] |
| (Context length)^2 | 7.33 | 1227.0 | **4.1E-13** | 4.1E-05 | [3.0E-05, 5.2E-05] |
| Layer | 6.66 | 1227.0 | **4.1E-11** | 1.2E-03 | [8.2E-04, 1.5E-03] |
| (Layer)^2 | -4.31 | 1227.0 | **1.8E-05** | -2.0E-05 | [-2.8E-05, -1.1E-05] |
| Layer: Context_length | -4.77 | 1227.0 | **2.1E-06** | -2.0E-05 | [-2.8E-05, -1.2E-05] |

The ‘Linguistic voxels vs. Cross-modal voxels’ comparisons include both a random intercept and random slopes (1 + type | participant). The ‘Bimodal voxels vs. Production voxels’ comparisons include both a random intercept and random slopes (1 + context + type | participant). The ‘Bimodal voxels vs. Comprehension voxels’ comparison includes a random intercept and a random slope (1 + context | participant).

**Supplementary Table 4 | The number of fMRI data samples (or runs) collected per session.**

| Participant | Ses. 1 | Ses. 2 | Ses. 3 | Ses. 4 |
|---|---|---|---|---|
| P1 | 2,580 (6) | 3,870 (9) | 1,290 (3) | 3,870 (9) |
| P2 | 3,010 (7) | 3,870 (9) | 3,870 (9) | N/A |
| P3 | 3,440 (8) | 3,870 (9) | 4,300 (10) | N/A |
| P4 | 3,010 (7) | 3,440 (8) | 3,870 (9) | 1,290 (3) |
| P5 | 3,010 (7) | 3,870 (9) | 3,870 (9) | N/A |
| P6 | 3,010 (7) | 3,440 (8) | 3,870 (9) | 1,290 (3) |
| P7 | 3,010 (7) | 3,870 (9) | 3,010 (7) | 1,720 (4) |
| P8 | 3,010 (7) | 3,870 (9) | 3,870 (9) | 860 (2) |

The number in brackets indicates the number of runs.

**Supplementary Table 5 | Topic list used in the dialogue experiment.**

| Theme | Topics |
|---|---|
| *self-introduction* | self-introduction |
| *plans, schedules, dreams* | today, this week, distant future |
| *people* | family, friends, liked individuals, disliked individuals |
| *spatial direction/explanation* | packing an eco-bag, layout and arrangement of a house |
| *place direction/explanation* | directions to the experiment location, etc. |
| *numerical operations* | arithmetic thinking and teaching methods |
| *consensus game* | escape from the jungle |
| *story creation game* | Rory's Story Cubes |
| *interview game* | understanding the other person's likes and dislikes |
| *abstract operations* | conceptually similar things; what it means to be human |
| *unrealistic imagination* | what would happen if everyone in the world had the same face? |
| *perspective* | how others or your future self perceive the current you |
| *emotions/metacognition* | regrets, dislikes, moments of happiness, moments of relaxation |
| *understanding others* | questions to ask the other person, encouraging them to talk |
| *memory recall* | impressions and questions about the experiment |
| *reasoning using everyday knowledge* | a TV program the other person watched: a bus route that operates only once a year; where is it? why does it continue despite being unprofitable? |
| *experience sharing* | explanation or recommendation of books, videos, etc. |
| *geography/values* | cities or regions where you live(d) or want to live |
| *science/logical problems* | differences between astrology and astronomy, causes of colds, correlation with ice cream sales, reasons for increased sales |
| *self-justification* | things you want to quit but can't |
| *storytelling* | recalling fairy tales and analyzing their narrative structure |
| *ethics* | drunk driving, age restrictions on alcohol consumption, jaywalking, unlicensed driving, COVID-19 vaccine prioritization, insurance coverage |
| *expectations* | marriage partner, future life |
| *discounting principle* | would you prefer a three-month bonus today or in one year? Marshmallow Test |
| *opinion expression* | appropriate formats for classes and tests (active learning/lecture, online/in-person) |
| *language expression* | conveying difficult messages: how to decline a request or point out a mistake |
| *dialogue management* | free topic |
