# Supplementary material for: Conversational content is organized across multiple timescales in the brain
Source: Nat Hum Behav. 2025 Jun 11;9(10):2066–78. doi: 10.1038/s41562-025-02231-4 (PMC12545197; doi:10.1038/s41562-025-02231-4)
Supplement: Supplementary file 2 — Reporting Summary [file 41562_2025_2231_MOESM2_ESM.pdf]

nature portfolio

Corresponding author(s): Shinji Nishimoto

Last updated by author(s): Apr 27, 2025

Reporting Summary

Nature Portfolio wishes to improve the reproducibility of the work that we publish. This form provides structure for consistency and transparency in reporting. For further information on Nature Portfolio policies, see our Editorial Policies and the Editorial Policy Checklist.

Statistics

For all statistical analyses, confirm that the following items are present in the figure legend, table legend, main text, or Methods section.

n/a	Confirmed	
☐	☒	The exact sample size (n) for each experimental group/condition, given as a discrete number and unit of measurement
☐	☒	A statement on whether measurements were taken from distinct samples or whether the same sample was measured repeatedly
☐	☒	The statistical test(s) used AND whether they are one- or two-sided<br>*Only common tests should be described solely by name; describe more complex techniques in the Methods section.*
☐	☒	A description of all covariates tested
☐	☒	A description of any assumptions or corrections, such as tests of normality and adjustment for multiple comparisons
☐	☒	A full description of the statistical parameters including central tendency (e.g. means) or other basic estimates (e.g. regression coefficient) AND variation (e.g. standard deviation) or associated estimates of uncertainty (e.g. confidence intervals)
☐	☒	For null hypothesis testing, the test statistic (e.g. F, t, r) with confidence intervals, effect sizes, degrees of freedom and P value noted<br>*Give P values as exact values whenever suitable.*
☒	☐	For Bayesian analysis, information on the choice of priors and Markov chain Monte Carlo settings
☒	☐	For hierarchical and complex designs, identification of the appropriate level for tests and full reporting of outcomes
☐	☒	Estimates of effect sizes (e.g. Cohen's d, Pearson's r), indicating how they were calculated

Our web collection on statistics for biologists contains articles on many of the points above.

Software and code

Policy information about availability of computer code

Data collection	Siemens MAGNETOM Prisma (syngo MR E11) and Siemens MAGNETOM Prisma Fit (syngo MR E11).
Data analysis	The code supporting the findings of this study is available on GitHub: https://github.com/yamashita-lang/dialogue.<br>Initial transcription was performed using Microsoft Azure Speech-to-Text (Speech SDK version 1.16.0; https://learn.microsoft.com/en-us/azure/ai-services/speech-service/).<br>fMRI data preprocessing was conducted using MATLAB (R2019b, MathWorks Inc.) in combination with SPM8 (https://www.fil.ion.ucl.ac.uk/spm/).<br>Banded ridge regression was implemented in Python 3 using the Himalaya package (version 0.4.2; https://gallantlab.org/himalaya/) with PyTorch (version 2.1.2+cu118; https://pytorch.org/).<br>Language data were processed using various tools, including Japanese GPT models (https://huggingface.co/rinna/japanese-gpt-neox-3.6b-instruction-sft; https://huggingface.co/rinna/japanese-gpt-neox-3.6b) and code adapted from https://github.com/mtoneva/brain_language_NLP.<br>Data analysis was carried out using NumPy (version 1.26.4; https://numpy.org/), SciPy (version 1.12.0; https://scipy.org/), and Scikit-learn (version 1.4.0; https://scikit-learn.org/).<br>Cortical surface visualizations were generated using FreeSurfer (version 6.0; https://surfer.nmr.mgh.harvard.edu/) and Pycortex (version 1.2; https://github.com/gallantlab/pycortex).<br>Plots were created with the Matplotlib (version 3.8.2; https://matplotlib.org/) and seaborn (version 0.13.2; https://seaborn.pydata.org/).<br>Linear mixed-effects modeling was conducted in R (version 4.3.3; https://cran.r-project.org) using the lmerTest package (version 3.1-3; https://cran.r-project.org/web/packages/lmerTest/index.html).

For manuscripts utilizing custom algorithms or software that are central to the research but not yet described in published literature, software must be made available to editors and reviewers. We strongly encourage code deposition in a community repository (e.g. GitHub). See the Nature Portfolio guidelines for submitting code & software for further information.

Data

Policy information about availability of data

All manuscripts must include a data availability statement. This statement should provide the following information, where applicable:

- Accession codes, unique identifiers, or web links for publicly available datasets
- A description of any restrictions on data availability
- For clinical datasets or third party data, please ensure that the statement adheres to our policy

MRI data and preprocessed stimulus features used in the current study are available via OpenNeuro at https://openneuro.org/datasets/ds004669.
The Destrieux Atlas can be accessed via the FreeSurfer software package (https://surfer.nmr.mgh.harvard.edu/fswiki/CorticalParcellation).
The Corpus of Everyday Japanese is available from the National Institute for Japanese Language and Linguistics (https://www2.ninjal.ac.jp/conversation/cejc-monitor.html).
Because the free-form conversations include complex details that could reveal participants' identities, the raw speech data—after removal of personal identifiers—will be provided only to researchers who (i) contact the corresponding author (S.N.) and (ii) sign a data-sharing agreement that complies with the regulations of the relevant ethics committees and with applicable privacy laws.

Research involving human participants, their data, or biological material

Policy information about studies with human participants or human data. See also policy information about sex, gender (identity/presentation), and sexual orientation and race, ethnicity and racism.

Reporting on sex and gender	The participants were selected to include male and female. Gender was determined based on self-reporting. Since these variables were outside of this study, they were not considered in our analysis.
Reporting on race, ethnicity, or other socially relevant groupings	Racial or ethnic information was not collected. Participants self-reported to be a native speaker of the language (Japanese) used in the experiment.
Population characteristics	Participants (3 female, 5 male) between 20 and 23 years of age, with normal hearing, normal or corrected-to-normal vision, without history of diagnosis of language or hearing diseases.
Recruitment	Participants were recruited through social networking sites (Twitter). They were undergraduate students from the nearby area. There was no self-selection bias.
Ethics oversight	The experiment was approved by the Ethical Committee of National Institute of Information and Communications. Technology.

Note that full information on the approval of the study protocol must also be provided in the manuscript.

Field-specific reporting

Please select the one below that is the best fit for your research. If you are not sure, read the appropriate sections before making your selection.

☒ Life sciences ☐ Behavioural & social sciences ☐ Ecological, evolutionary & environmental sciences

For a reference copy of the document with all sections, see nature.com/documents/nr-reporting-summary-flat.pdf

Life sciences study design

All studies must disclose on these points even when the disclosure is negative.

Sample size	Data analysis and statistics were examined and confirmed for each participant separately. The sample size of the test data (at least 860 samples) was determined to perform proper predictive and statistical analysis for encoding models and to match our prior attempts (e.g., Nakai and Nishimoto, 2020 Nature Communications).
Data exclusions	Some data were excluded from the analysis if only one run of fMRI data was available within one session (fourth session of participant P2 and fourth session of participant P5).
Replication	Encoding models were fit and evaluated for each participant, and the results were consistent across the 8 participants. The sample size is comparable to previous studies using voxel-wise encoding model (e.g., Nakai and Nishimoto, 2020 Nature Communications).
Randomization	Randomization was not relevant to this study as participants were not allocated into experimental groups.
Blinding	Blinding was not relevant to this study as participants were not allocated into experimental groups.

Reporting for specific materials, systems and methods

We require information from authors about some types of materials, experimental systems and methods used in many studies. Here, indicate whether each material, system or method listed is relevant to your study. If you are not sure if a list item applies to your research, read the appropriate section before selecting a response.

Materials & experimental systems

n/a	Involved in the study
☒	☐ Antibodies
☒	☐ Eukaryotic cell lines
☒	☐ Palaeontology and archaeology
☒	☐ Animals and other organisms
☒	☐ Clinical data
☒	☐ Dual use research of concern
☒	☐ Plants

Methods

n/a	Involved in the study
☒	☐ ChIP-seq
☒	☐ Flow cytometry
☐	☒ MRI-based neuroimaging

Magnetic resonance imaging

Experimental design

Design type: Building voxel-wise encoding models using task-evoked brain activity (Nishimoto et al., 2011 Current Biology).

Design specifications: The experiment was conducted in three or four separate fMRI sessions. Each session collected 2 to 10 runs. The total of 27 runs were acquired across the sessions. A single run consisted of 430 seconds.

Behavioral performance measures: Behavioral performance was not measured quantitatively. The experimenter who engaged in the conversation judged based on whether the participant continued the conversation.

Acquisition

Imaging type(s): functional, structural.

Field strength: 3T.

Sequence & imaging parameters: Functional data: A multiband gradient echo-planar imaging sequence (TR = 1,000 ms, TE = 30 ms, flip angle = 60°; voxel size = 2 × 2 × 2 mm3, matrix size = 96 × 96, 72 axial slices, FOV = 192 × 192 mm2, multiband factor = 6).

Structural data: T1-weighted MPRAGE (TR = 2530 ms, TE = 3.26 ms, flip angle = 9°, voxel size = 1 × 1 × 1 mm3, matrix size = 256 × 256, 256 axial slices, FOV = 256 × 256 mm2).

Area of acquisition: A whole-brain scan was used.

Diffusion MRI ☐ Used ☒ Not used

Preprocessing

Preprocessing software: SPM8 (motion correction) and FreeSurfer 5.3.0 (anatomical registration, cortical surface reconstruction, cortical segmentation, and subcortical segmentation).

Normalization: Data were not normalized. Data for each participant were analyzed individually.

Normalization template: The data were not normalized.

Noise and artifact removal: Motion correction (6DOF) was performed by aligning all of the EPI data to the first image from the first scan for each subject. For each voxel, responses were normalized by subtracting the mean response across all time points, and trend was removed using a median filter (120-s time window). These processes were performed using in-house MATLAB codes (Cukur et al., 2016 The Journal of Neuroscience). No spatial smoothing procedure was performed.

Volume censoring: No censoring was performed and all data were used for the study.

Statistical modeling & inference

Model type and settings: Multivariate, predictive. Feature-based encoding models were built using the training data, and the modeling accuracy was examined by using the held-out test data. Leave-one-session-out cross-validation was performed to estimate average modeling accuracy across the sessions.

Effect(s) tested | Prediction performance was calculated by Pearson correlation coefficient between measured and predicted BOLD responses for each voxel.

Specify type of analysis: ☒ Whole brain ☐ ROI-based ☐ Both

Statistic type for inference (See Eklund et al. 2016) | voxel-wise.

Correction | False-discovery rate (FDR) correction (Benjamini and Hochberg, 1995).

Models & analysis

n/a	Involved in the study
☒	☐ Functional and/or effective connectivity
☒	☐ Graph analysis
☐	☒ Multivariate modeling or predictive analysis

Multivariate modeling and predictive analysis | We built voxel-wise encoding models (Naselaris et al., 2011 NeuroImage; Nishimoto et al., 2011 Current Biology) to explain the BOLD responses using speech-related features. Linguistic features of each stimulus utterance were extracted from a pre-trained GPT language model. Model weights were estimated using a L2-regularized linear regression procedure (Huth et al., 2012 Neuron) for training data (6,880 to 10,750 samples). The regularization parameter was optimized via 5-fold cross validation using the training data. The prediction accuracy of each voxel model was quantified by a Pearson's correlation coefficients between the measured and the predicted BOLD responses for test data (860 to 4,300 samples).
